# Photonic Eigenmodes of 2D Cylindrical Cholesteric Liquid Crystal Resonators

**DOI:** 10.1021/acsphotonics.5c01294

**Published:** 2025-09-26

**Authors:** Urban Mur, Jaka Zaplotnik, Martin Horvat, Igor Muševič, Miha Ravnik

**Affiliations:** † Faculty of Mathematics and Physics, 61790 University of Ljubljana, Jadranska ulica 19, Ljubljana SI-1000, Slovenia; ‡ Jozef Stefan Institute, Jamova cesta 39, Ljubljana SI-1000, Slovenia; § Department of Engineering Science, University of Oxford, Parks Road, Oxford OX1 3PJ, United Kingdom

**Keywords:** cholesteric liquid crystal, resonator, optical eigenmode, spectrum

## Abstract

Cholesteric liquid crystals (CLCs) are birefringent materials with a helical molecular orientation that enables the selective reflection of circularly polarized light, making them valuable for various optical applications. While extensively studied in planar geometries, their optical properties in cylindrical and droplet-shaped confinements remain less understood. This article numerically investigates photonic eigenmodes in 2D cylindrical CLC resonators with concentric layered and spiral configurations. We demonstrate that the interplay of cylindrical confinement and cholesteric helicity gives rise to distinct optical modes: (i) Bragg-like modes, (ii) central defect modes, and (iii) whispering gallery modes at the boundary or within the bulk. These findings connect the well-known behavior of 1D CLC layers with more complex 2D cylindrical and 3D spherical systems and provide insight into the polarization-dependent mode structure in anisotropic media. The results have implications for designing advanced CLC-based photonic elements such as soft-matter-based lasers and spherical reflectors.

## Graphical abstract

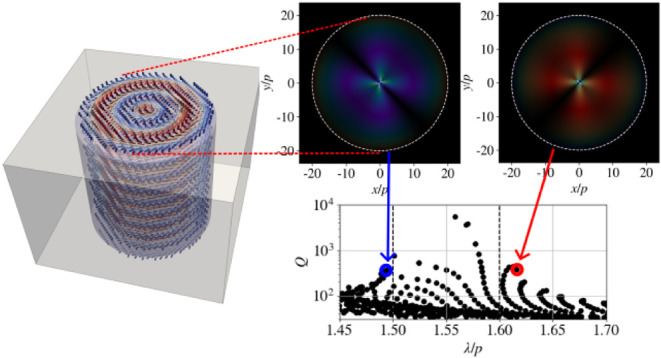

## 1 Introduction

Cholesteric liquid crystals (CLCs) are birefringent optical materials capable of selectively reflecting light, due to their periodic, spontaneously formed helical molecular organization.^1–7^ The selective reflection creates a photonic band gap for circularly polarized light with the same handedness as the cholesteric helix and propagating along the helical axis. This distinct optical characteristic makes CLCs interesting for various applications, such as filters,^8–10^ isolators,^11^ light shutters,^12^ and diffractive optical devices.^13^ Importantly, the self-assembled helical structure of CLCs can function as an optical resonator, supporting tunable band-edge lasing^14–28^ and defect lasing.^29,30^

Most of these applications have been realized in planar cholesteric cells, where the material is confined by well-defined planar boundary conditions on the top and bottom surfaces and the lateral size of the cell is much larger than its thickness. In such configurations, the orientation of the CLC optical axis essentially exhibits variations only along the helical axis, i.e., along one spatial direction. The optical properties of such planar CLC geometries have been extensively explored both experimentally and theoretically, allowing for a comprehensive understanding of their spectra and well-defined optical modes.^31^ Knowledge of optical modes proves to be crucial in more advanced applications, for example, optical switching of lasing pulses.^32^

However, CLCs have been utilized for optical applications in more complex geometries. When CLC is confined into a cylindrical cavity, it can self-organize into various structures, depending on the material parameters and surface anchoring^33–35^ one of them being a radially twisted structure, with the twist axis perpendicular to the boundary of the cavity, which is stable for high chiralities (i.e., high radius-to-pitch ratios). Such a structure was observed experimentally in nano- and bio-colloidal CLC^36,37^ and used in stimuli-responsive CLC elastomer fibers.^38^ CLC in cylindrical confinement was also used for tunable photonic crystal fibers.^39,40^ CLC-infiltrated fibers with other shapes have been studied as well.^41^

Additionally, lasing from CLC-based cavities has also been demonstrated in 3D geometries. Experimental studies have shown that CLC droplets can function as Bragg resonators, emitting light from their centers^42^ and also in the form of rings,^43^ as whispering gallery mode (WGM) lasers^44^ and as manipulators for lasing modes of Fabry–Pérot resonators.^45^ CLC droplet microlasers have been employed in multicolor lasers^46^ and used as temperature sensors,^47,48^ biodegradable sensors,^49^ wearable skin sensors^50^ and light sources for digital holography.^51^ Magnetically transportable lasers,^52^ a degree of control on lasing of different modes^53^ and simultaneous multiwavelength lasing^54^ were also reported in shell-structured CLC droplets. Besides lasing, CLC droplets have been widely used as color-changing materials for sensing applications^55,56^ and tunable spherical reflectors.^57,58^

Despite all of the above-mentioned applications in optics and photonics, the understanding of optical modes in CLC cylinders, and especially in droplets, remains rather limited. There have been theoretical studies on the propagation of light along CLC fibers with the helical axis parallel to the axis of the cylindrical confinement.^59^ Passive optical modes for lasing have been studied using the Finite Difference Frequency Domain numerical method in 1D cholesterics^31^ and 3D nematic structures.^60,61^ Photonic eigenmodes of CLC droplets inserted in a Fabry–Pérot resonator have been calculated in 2D using the Finite Element Method; however, the periodic helical twisting was approximated by alternating isotropic layers with different refractive indices.^45^ Similarly, radially oscillating modes and WGMs have been analyzed theoretically and numerically in isotropic cylindrical layered structures^62^ and isotropic spherical Bragg onion resonators.^63–65^ While the understanding of modes in isotropic layered systems can be useful, these do not fully capture the optical properties of birefringent cholesteric layers, where polarization-dependent reflections and mode coupling due to anisotropy occur.

This paper demonstrates the photonic eigenmodes of 2D cylindrical cholesteric liquid crystal resonators with a concentric layered and spiral structure by using numerical modeling. Specifically, we show that a combination of cylindrical confinement and a cholesteric helical birefringent optical profile leads to spatially and structurally very different photonic modes, i.e., (i) Bragg-like CLC edge modes, (ii) defect modes occurring at the center of the cylindrical structure, and (iii) whispering gallery modes, which can occur at the boundary of the resonator or at the CLC layered structure in the bulk. More broadly, the results also bridge the gap between the well-explored optical properties of 1D CLC layers and the more complex 3D CLC droplets, as the considered 2D CLC cylindrical profiles directly correspond also to the two main cross sections—concentric layered and spiral—of 3D CLC droplets with the radial spherical/spherulitic director structure.^66^ More generally, the understanding of the different modes has interesting potential for use in the development and design of optical elements such as lasers and soft matter-based quantum light sources.

## 2 Methods

We explore the passive photonic eigenmodes of a cholesteric liquid crystal in cylindrical confinement by using the Finite Difference Frequency Domain (FDFD) method.^67,68^ In the calculations, we assume that the total electric field is a superposition of multiple harmonic modes with well-defined frequencies

1 $$E\left(r,t\right)=\underset{\mu }{\sum }\Psi_{\mu }\left(r\right)e^{-i\omega_{\mu }t}$$

where **Ψ**_μ_ are the electric field profiles—i.e., the optical modes—and ω_μ_ are the corresponding frequencies.

Such a harmonic field profile is inserted into Maxwell’s equations, which give the so-called Master equation for calculating the photonic modes as

2 $$\nabla \times \nabla \times \Psi_{\mu }\left(r\right)={\left(\frac{\omega_{\mu }}{c_{0}}\right)}^{2}\underset{\_}{\varepsilon \left(r\right)}\Psi_{\mu }\left(r\right)$$

where *c*_0_ is the speed of light in vacuum and $\underset{\_}{\varepsilon \left(r\right)}$ is the spatially dependent relative dielectric permittivity tensor that contains the information about the birefringent cholesteric LC profile and the geometry of the system. Mathematically, eq 2 represents the eigenproblem of our system with eigenvectors being the electric field profiles **Ψ**_μ_ and eigenvalues of their corresponding frequencies ω_μ_. For liquid crystal, the components of the relative dielectric permittivity tensor ϵ_*kl*_ can be related to the cholesteric liquid crystal director field as

3 $$\varepsilon_{kl}=n_{o}^{2}\delta_{kl}+\left(n_{e}^{2}-n_{o}^{2}\right)n_{k}n_{l}$$

where *k*,*l* ∈ (*x*, *y*, *z*), δ_*k*,*l*_ is the Kronecker delta, *n*_*k*_ are components of the director field ***n*** (***r***), and *n*_*o*_ and *n*_*e*_ are the ordinary and the extraordinary refractive indices of the liquid crystal, respectively. We assume that the cylindrical liquid crystal region is surrounded by an isotropic optical matrix, characterized by the isotropic dielectric tensor $\varepsilon_{kl}=n_{i}^{2}\delta_{kl}$ where *n*_*i*_ is the isotropic refractive index of the surrounding isotropic matrix. We assume no absorption; therefore, the dielectric tensor is real everywhere in the simulation volume, except in the Perfectly Matched Layer (PML) region, which is used to simulate open boundary conditions. PML is realized by placing an artificial material at the boundaries. The material has a certain complex permittivity and permeability such that it is absorbing and analytically reflectionless.^68^ Consequently, the calculated eigenvectors (i.e., electric field profiles) and eigenvalues (i.e., frequencies) are complex. We characterize the calculated eigenmodes in terms of *Q*-factors

4 Qμ=|Re(ωμ)2Im(ωμ)|

which give the ratio between the energy stored in the resonator and the energy lost in the absorbing boundary. The *Q*-factors are a measure of the quality of the resonance—a higher *Q*-factor corresponds to a narrower spectral line and therefore better resonance—and are related to the lifetimes of the modes. The spectra that we show visualize the modes as dots on the *Q*_μ_(λ_μ_/*p*) plot, where

5 λμp=2πc0Re(ωμ)p

is the dimensionless wavelength of a given mode and *p* is the cholesteric pitch—the distance along the helical axis of the cholesteric liquid crystal that corresponds to a rotation of the director of 360°.

The Master equation (eq 2) is solved using iterative eigensolvers from the PETSc toolkit,^69^ as inspired by ref. 68. The shift-invert transformation is employed to efficiently compute eigenvalues near a specified target frequency or wavelength. The eigenvalue problem is solved using the SLEPc eigenvalue Problem Solver with its default algorithm, the Krylov–Schur method, which is described in detail in the SLEPc Technical Report STR-7.^70^ Unless stated differently, we calculate the spectrum for a radius of the cylindrical resonator *R* = 20*p* and a resolution of 20 simulation grid points per pitch. The dimension of the matrix required to solve the considered 3D eigenproblem is *N* = 2 · 3 · *N*_*x*_ *N*_*y*_, where 3 corresponds to the electric field components, 2 accounts for the real and imaginary parts, and *N*_*i*_ are the system dimensions in grid points. The number of matrix elements *N* thus scales approximately with *R* ^4^, where *R* is the cylinder radius; however, the matrix is sparse, which eases the computation. Approximately 10–30 modes can be identified in a single run in a narrow range around a selected initial eigenfrequency approximation. The full spectrum—wavelengths and *Q*-factors of the modes—is obtained by sweeping the frequency range of interest using multiple runs with different initial eigenfrequency approximations.

## 3 Geometry of the System

The spatially varying optical axis of the birefringent 2D cylindrical CLC cavity with concentric CLC layers, referred to as the concentric structure, is shown by the nematic director field in Figure 1A–C and can be described within the radius of the cylinder *R* by the following profile:

6 $$n\left(r,\phi \right)=\left(n_{x}\left(r,\phi \right),n_{y}\left(r,\phi \right),n_{z}\left(r,\phi \right)\right)=\left(\sin\phi \;\sin\left(\frac{2\pi r}{p}\right),-\;\cos\phi \;\sin\left(\frac{2\pi r}{p}\right),\cos\left(\frac{2\pi r}{p}\right)\right)$$

where $r=\sqrt{x^{2}+y^{2}}$ , ϕ = atan2­(*y*, *x*) is the polar angle and *p* is the cholesteric pitch—the distance along the helical axis of the cholesteric liquid crystal that corresponds to a rotation of the director of 360°. The cylinder is assumed to be surrounded by an isotropic dielectric material with refractive index *n*_*i*_. Such a director profile encompasses the same helical structure—now in the *radial* direction—as is normally observed in 1D CLC resonators:^31^

7 $$n\left(r,\phi =0\right)=\left(0,-\;\sin\left(\frac{2\pi r}{p}\right),\;\cos\left(\frac{2\pi r}{p}\right)\right)$$

Note that the director profile is continuous also in the center of cylinder ***n*** (*r* = 0, ϕ) = (0, 0, 1), leading to a continuous dielectric profile, calculated by eq 3. While the simulations take into account all three spatial components of the director and electromagnetic fields, they are conducted in two dimensions—essentially a slice of a 3D schematic resonator, which is shown in Figure 1A. The actual 2D simulation box is shown in Figure 1B. A Perfectly Matched Layer (PML) absorbing boundary condition is used to simulate open boundaries in all four directions.

**Figure 1:**
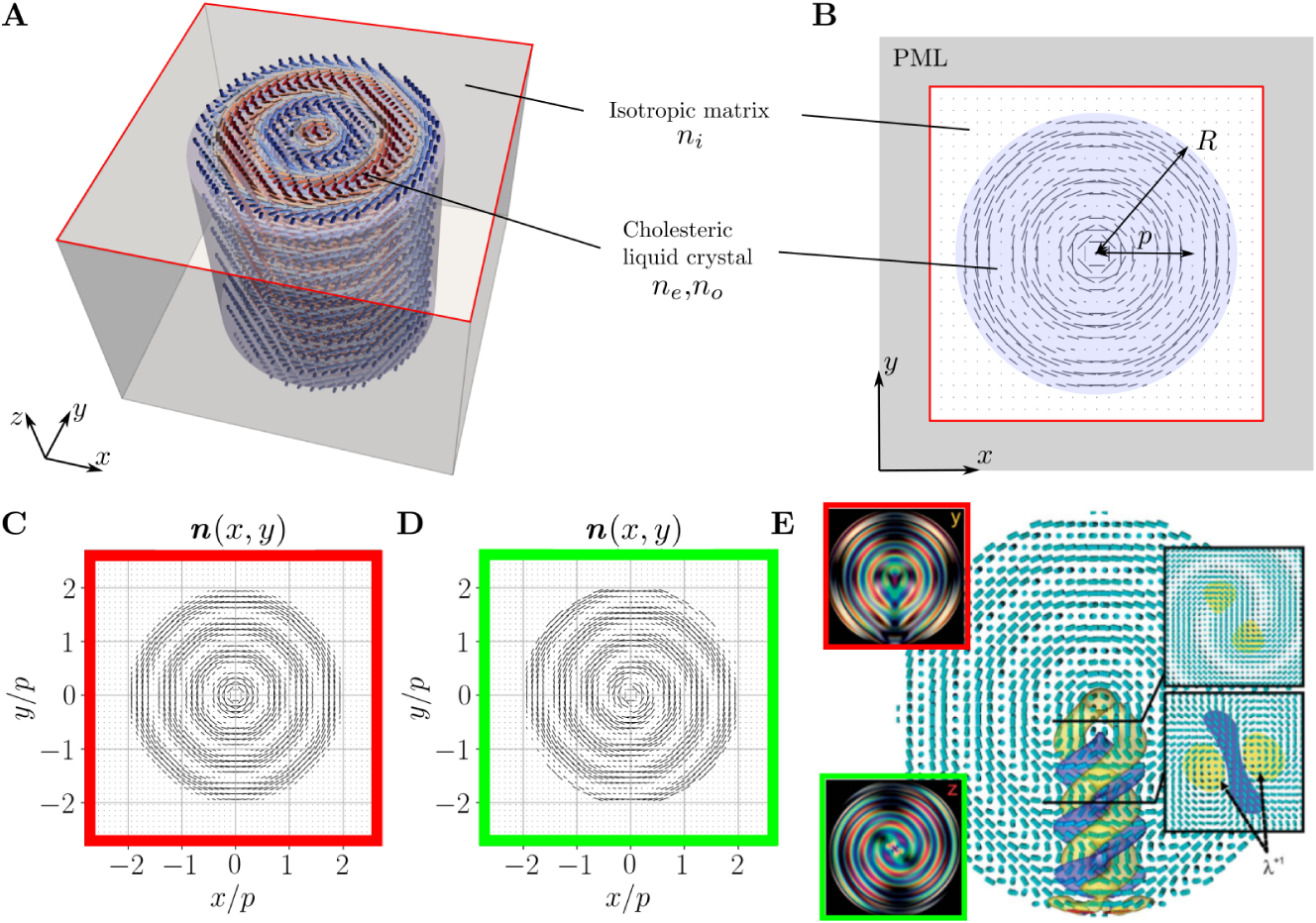
Geometries of the relevant CLC resonators. (**A**) 3D schematic of the cylindrical CLC resonator. (**B**) Schematic of the 2D simulation box. CLC cylindrical layers with ordinary and extraordinary refractive indices *n*_*o*_ and *n*_*e*_, respectively, pitch *p* and radius *R* are surrounded by the isotropic material with refractive index *n*_*i*_. The simulation box is surrounded by the Perfectly Matched Layers (PML) to model open boundary condition. The considered CLC profile of (**C**) concentric and (**D**) spiral director fields. (**E**) Director field of the RSS structure in a typical cholesteric droplet with Polarized Optical Microscopy (POM) images of two characteristic cross sections. Reproduced from ref. 66 with permission from the Royal Society of Chemistry. Note how our simulated profile from (**C**) approximates the cross section with the concentric layers, marked in red in panel (**E**), and how the simulated profile in (**D**) approximates the cross section with spiral layers, marked in green in panel (**E**). Blue and yellow colors visualize the isosurfaces of the splay-bend parameter, which characteristically marks the twisted disclination defect lines. Those defect lines are omitted from our 2D calculations.

We note here that the chosen geometry of the system—i.e., the director field—also well captures the main optical variability of the director field in selected cross sections of 3D (!) spherical CLC droplets. It has been shown that typically the most stable structure in a CLC droplet with planar boundary conditions is the radial spherical structure (RSS, also known as the spherulitic texture or the Frank–Pryce model), which exhibits concentric CLC layers with the helical axis oriented radially and a radial escaped disclination defect line extending from the center to the droplet’s boundary^66,71^ (see Figure 1C–E). Both cross sections of the 3D droplet are in fair relation to the profile that we consider in our CLC cylindrical resonators, with the exception of the disclination defect line, which is not present in the 2D geometries. Therefore, effectively, the photonic response of our considered 2D CLC cylindrical resonators can provide selected insight into the photonic response of 3D CLC droplets.

## 4 Results

### 4.1 Spectrum of a 2D Cylindrical CLC Resonator

The frequencies and *Q*-factors—spectra—of eigenmodes of a 2D cylindrical CLC resonator calculated by the FDFD method (see Section 2) are shown in Figure 2. Specifically, we focus on the part of the spectrum in the proximity of the 1D photonic band gap, which occurs between λ/*p* = *n*_*o*_ and λ/*p* = *n*_*e*_ and where the interesting optical phenomena are expected due to selective light reflection on cholesteric layers. In general, the spectra are more complicated than the spectra of 1D CLC layers^31^ and no photonic band gap is observed. Figure 2A shows the spectra for *n*_*o*_ = 1.5, *n*_*e*_ = 1.6 and three different values of radii of the CLC cylinder in the units of pitch *p* (*R*/*p* = 5, *R*/*p* = 10, and *R*/*p* = 20). We notice that by increasing the number of pitches in the radius, which essentially increases the number of cholesteric layers in the cylinder, the *Q*-factors of the modes generally increase, which is in line with results for 1D layers, where similar trends are observed for Bragg-like edge modes. In addition, the clustering of the modes at the frequencies that coincide with the edges of the 1D frequency band gap becomes more prominent as we increase the number of layers in the cylinder (note the increased number of dots, marked by arrows, in the vicinity of the vertical dashed lines for larger *R*/*p*).

**Figure 2:**
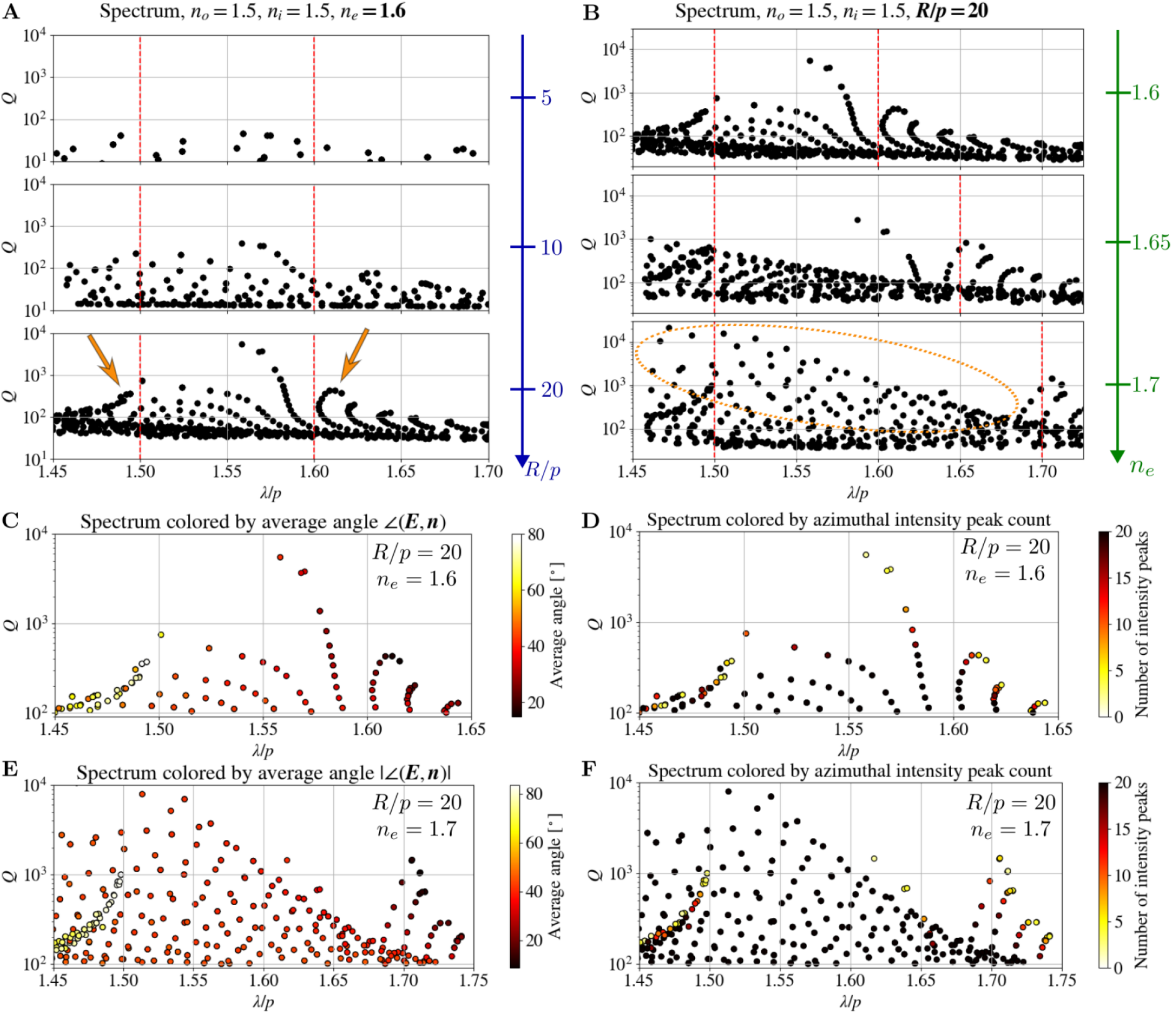
Spectra of the 2D cylindrical CLC resonator. Spectra are shown for (**A**) different radii measured in units of cholesteric pitches and (**B**) different values of the extraordinary refractive index *n*_*e*_. Vertical red dashed lines mark the theoretical edges of the 1D band gap at λ/*p* = *n*_*o*_ and λ/*p* = *n*_*e*_. Each point represents one eigenmode for the selected parameters. Arrows in panel (**A**) mark the clusters of modes in the vicinity of the 1D band gap edges, which become more significant for larger *R*/*p*. Dotted ellipses in panel (**B**) roughly mark the new family of modes that emerges on the blue (i.e., shorter wavelength) side of the spectrum as the birefringence is increased. The 3 subplots in each panel share the same *y*-axis. (**C–F**) Spectra of the resonator with *R*/*p* = 20 and (**C,D**) *n*_*e*_ = 1.6 or (**E,F**) *n*_*e*_ = 1.7, colored by (**C,E**) the intensity-weighted average angle between the director and electric field and (**D,F**) electric field intensity maxima in the azimuthal direction.

Figure 2B shows the spectra for *R*/*p* = 20 and three different values of the extraordinary refractive index *n*_*e*_ (*n*_*e*_ = 1.6, *n*_*e*_ = 1.65, and *n*_*e*_ = 1.7), while keeping the ordinary refractive index fixed at *n*_*o*_ = 1.5. By increasing the birefringence, we still observe clustering of the modes at the frequencies corresponding to the edges of the 1D band gaps, which also widen. Additionally, by increasing the *n*_*e*_, a new group of modes with high *Q*-factors, roughly marked with dotted ellipse in Figure 2B, emerges on the side of the spectrum corresponding to shorter wavelengths.

The structure of the spectra can be roughly explained by categorizing the modes into four distinct groups: Bragg-like edge modes, defect modes, WGMs, and hybrid WG-defect modes. Although there is no definitive boundary or metric to differentiate these modes (modes can even continuously transition between defect and edge modes in 1D under parameter variation!),^72^ they can be organized by examining their spatial electric field profiles, frequencies, alignment toward the director field and their changes when varying the material and geometrical parameters.

For instance, edge modes are typically characterized by their electric fields being either parallel or perpendicular to the CLC director field.^31^ To aid in identifying and distinguishing such mode families, we plot the spectra of modes with *Q* > 100, colored by the intensity-weighted average angle between the director field and the electric field of each mode, defined as

8 |∠(n,E)|=∑l(|Re(El)|2·∠(nl,El))∑l|Re(El)|2

where *l* indexes the computational mesh nodes. The result for the resonator with *R*/*p* = 20 and *n*_*e*_ = 1.6 or *n*_*e*_ = 1.7 is shown in Figure 2C,E. The visualization highlights important differences between the modes and emphasizes two families of modes near the 1D band gap edges.

In addition, we determine the number of intensity maxima in the azimuthal direction, a property closely related to the azimuthal mode number, as expected due to the system’s cylindrical symmetry. The spectra colored by the number of azimuthal intensity peak counts are shown in Figure 2D,F, revealing several distinct mode groups. Higher azimuthal mode numbers correspond to modes with more angular oscillations around the axis, and their presence reflects the underlying rotational invariance of the dielectric structure. Moreover, the number of azimuthal maxima can further aid in identifying different mode families, as modes with fewer intensity peaks (i.e., lower azimuthal mode numbers) are likely to originate from distinct physical mechanisms. It is important to note that the process of counting azimuthal maxima is not always straightforward. Identifying a consistent and robust peak-counting criterion for all the modes proves challenging, due to big differences in the widths of the peaks and as intensity variations may not be equally well defined. As a result, some inconsistencies in classification may occur, but nevertheless, significant differences between different types of modes can be observed.

Finally, to support the previously discussed parameters and visualizations, we directly compare 2D modes with the known modes in simpler systems, such as 1D CLC cavities or isotropic spheres, to provide further insights and classify the modes, as described in the following sections.

### 4.2 Bragg-like Edge Modes

Characteristic optical modes of a one-dimensional CLC resonator are edge modes^31^ as shown in Figure 3A. The resonator consists of a CLC layer with thickness *D* and helical profile of liquid crystal molecules, which is confined between two glass plates. Due to selective light reflection for circular polarization with the same handedness as the helix on the helical layered structure, the spectrum of such a 1D resonator exhibits a photonic band gap and the edge modes emerge at wavelengths close to the edges of the gap. Such a spectrum for a resonator with *D*/*p* = 20, *n*_*o*_ = 1.5, *n*_*e*_ = 1.6, and *n*_*i*_ = 1.5 is shown in Figure 3B. The basic mode that emerges at the red edge of the band gap (named as the R1 mode) is shown in Figure 3C. The light coming out of the resonator is nearly perfectly circularly polarized. Some of the most notable features of the edge modes observed in 1D CLC layers include values of wavelengths that are slightly shorter than λ/*p* = *n*_*o*_ or slightly longer than λ/*p* = *n*_*e*_, high *Q*-factors for modes with wavelengths near the band edges, and electric field profiles that follow a sinusoidal-like envelope within the bulk of the CLC.

**Figure 3:**
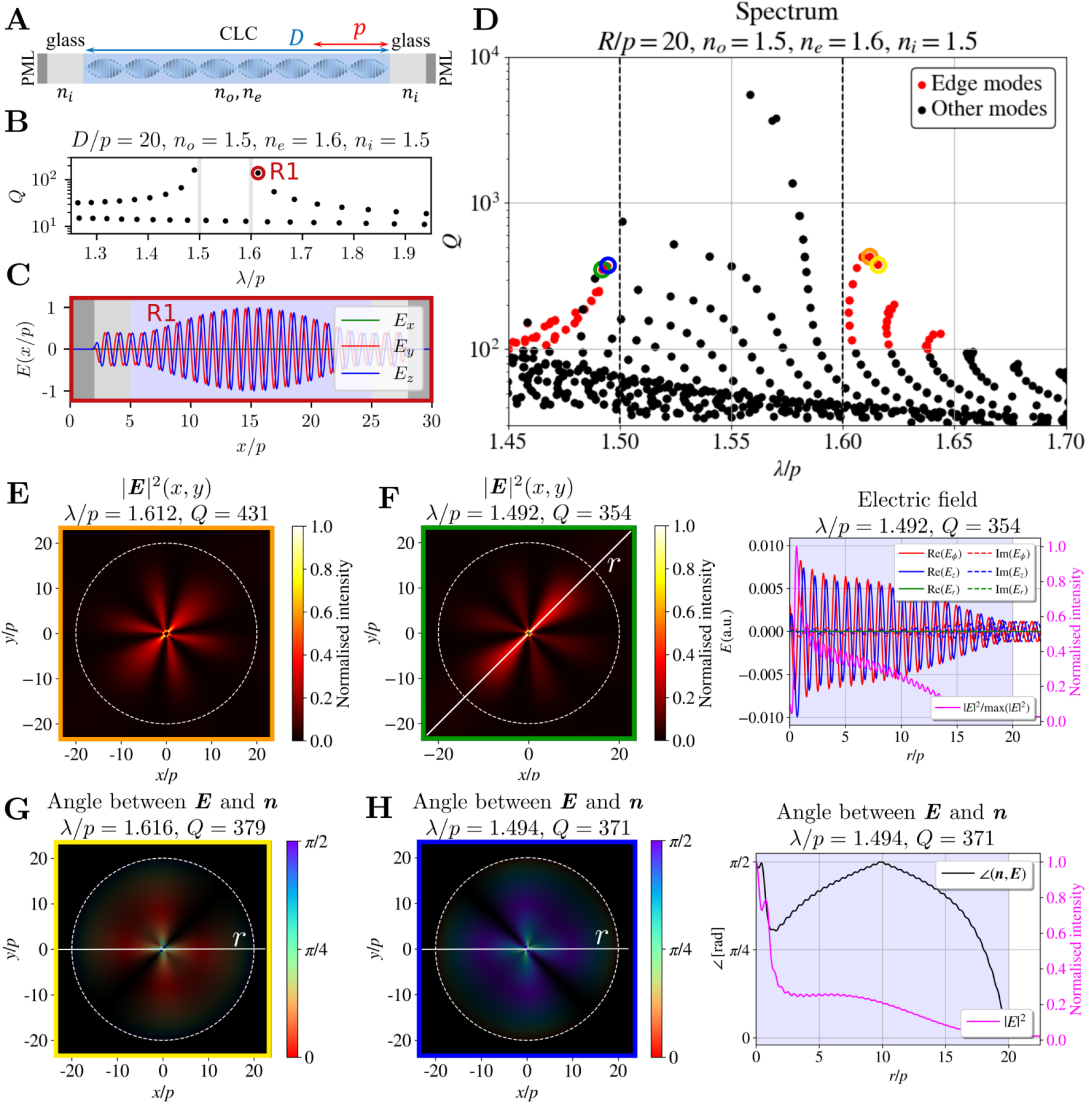
Edge modes in 1D and 2D cylindrical resonators. Edge modes in (**A**–**C**) 1D CLC and in (**D**–**H**) 2D CLC cylindrical resonators. (**A**) Schematic of the 1D CLC resonator, (**B**) spectrum of optical modes in the 1D CLC resonator with their corresponding *Q* factors, and (**C**) the electric field profile of the basic mode on the red side of the spectrum (the R1 mode). (**D**) Spectrum of a 2D cylindrical CLC resonator with marked edge modes. Simulation parameters are *R*/*p* = 20, *n*_*o*_ = 1.5, *n*_*e*_ = 1.6, and *n*_*i*_ = 1.5. (**E**) Electric field intensity | ***E*** |^2^ profile of the selected edge mode. (**F**) Electric field intensity profile with the marked direction of the intensity maximum (left) and the electric field and electric field intensity profiles in the radial direction at an angle marked with a white line (left) of another selected mode. (**G**,**H**) Color-coded angles between ***E*** and ***n*** for two modes. Brightness corresponds to the intensity. (**I**) Left: same angle as in (**H**) in the radial direction at the polar angle of intensity maximum (marked with a white line (**H**)). Right: electric field and electric field intensity profiles. Positions of all four modes in the spectrum are marked with the colors of the squares around the 2D profiles. In the right panel, the left *y*-axis corresponds to the electric fields, while the right *y*-axis shows the normalized electric field intensity (plotted in pink).

We observe effective edge modes also in the spectrum of the 2D cylindrical resonator with *R*/*p* = 20, *n*_*o*_ = 1.5, and *n*_*e*_ = 1.6, as shown in Figure 3D, which distinctly exhibit similar mode characteristics as the edge modes of 1D cholesterics. Specifically, their wavelengths lie just outside the theoretical 1D band gap, their intensity profiles follow a sinusoidal-like envelope, and they form an approximately constant angle with the director field. The identified edge modes with *Q* > 100 are marked with red dots in Figure 3D. The *Q*-factors of 2D edge modes are of the same order of magnitude as the *Q*-factors of the 1D edge modes for *D* = *R*. Slightly higher values are likely due to additional reflections in different halves of the diameter of the cylindrical resonator.

Selected edge modes are listed in Figure 3E–H. Figure 3E shows the electric field intensity of the mode with the highest *Q*-factor near the red edge. Figure 3F shows the electric field intensity | ***E*** |^2^ and profiles of the electric field components *E*_*x*_, *E_y_,* and *E*_*z*_ (which are in general complex) in the *xy*-plane (left) and in the radial direction at the polar angle of the intensity maximum (right), as marked with the white line in the left panel. From these profiles, we observe that the majority of the energy is concentrated in the bulk of the CLC, where the CLC layers are well-defined, and light is localized due to multiple Bragg-like reflections. The shape of the envelope of the standing wave in the resonator is similar to that for 1D CLC resonator modes. The *Q*-factors of 2D edge modes are of the same order of magnitude as the *Q*-factors of the 1D edge modes for *D* = *R*. Slightly higher values are likely due to additional reflections in different halves of the diameter of the cylindrical resonator. By observing the spectra in Figure 2, we can also note that the *Q*-factors of edge modes increase with both higher birefringence and larger droplet radius, consistent with results reported for 1D CLC layers.^31^

In addition to the field profiles, we also plot the angle between the electric field ***E*** and the director field ***n*** in Figure 3G,H. The far-left panels show the color-coded angle in the *xy*-plane, with the plot’s brightness corresponding to the intensity profile. The angle in the radial direction is displayed in the center panel, while the field profiles are shown in the right panel. Both edge modes exhibit a relatively constant angle between ***E*** and the ***n***, with the fields being predominantly parallel (red edge, Figure 3G) or perpendicular (blue edge, Figure 3H) to each other, similar to edge modes in 1D CLC layers.^31^

Due to the cylindrical symmetry of the resonator, the calculated electromagnetic edge modes are (nearly) degenerate. In ideal conditions, this means that multiple distinct field distributions correspond to the same complex eigenfrequency. However, in numerical simulations, degeneracy is typically lifted, primarily due to finite precision and numerical round-off errors (see Supporting Information for details). Despite slight numerical splitting, the complex frequencies of degenerate modes remain nearly identical—typically differing only by amounts on the order of the solver’s tolerance. This difference is much smaller than the typical separation between nondegenerate (distinct) modes (typically 1–2 orders of magnitude smaller), enabling reliable identification of degenerate pairs based on their close spectral proximity. Therefore, the degeneracy cannot be directly observed in the presented spectra, since all of the degenerate modes are seen as single dots.

Within the resonator, any complex linear combination of degenerate modes can be excited. This property leads to diverse physical manifestations, depending on how the degenerate modes are combined. In Figure 4 we show examples of two pairs of degenerate eigenvectors (panels (i) and (ii)) for two modes (Figure 4A,B), as computed with the FDFD method and their selected linear combinations. Panel (iii) shows the linear combination of the output modes with equal amplitude and the same phase, resulting in a standing wave that pulsates with a frequency of ω = *c*/λ. Panel (iv) presents linear combinations of the output modes with equal amplitude but a relative phase shift of π, giving rise to traveling (rotating) waves, circulating within the resonator. The time evolution of the real part of the electric field in panels (v) and (vi) further illustrates these distinctions, particularly highlighting the rotating behavior of the traveling wave mode. In simulations, for the degenerate modes, the symmetry of the finite difference mesh will determine the basis in which they will be represented. However, in reality, the excitation conditions (e.g., input phase, polarization, and location) will determine the specific linear combination of degenerate modes that will be realized in the resonator. Animations of the time evolution of various linear combinations of modes shown in Figure 4 are available in Supporting Information.

**Figure 4:**
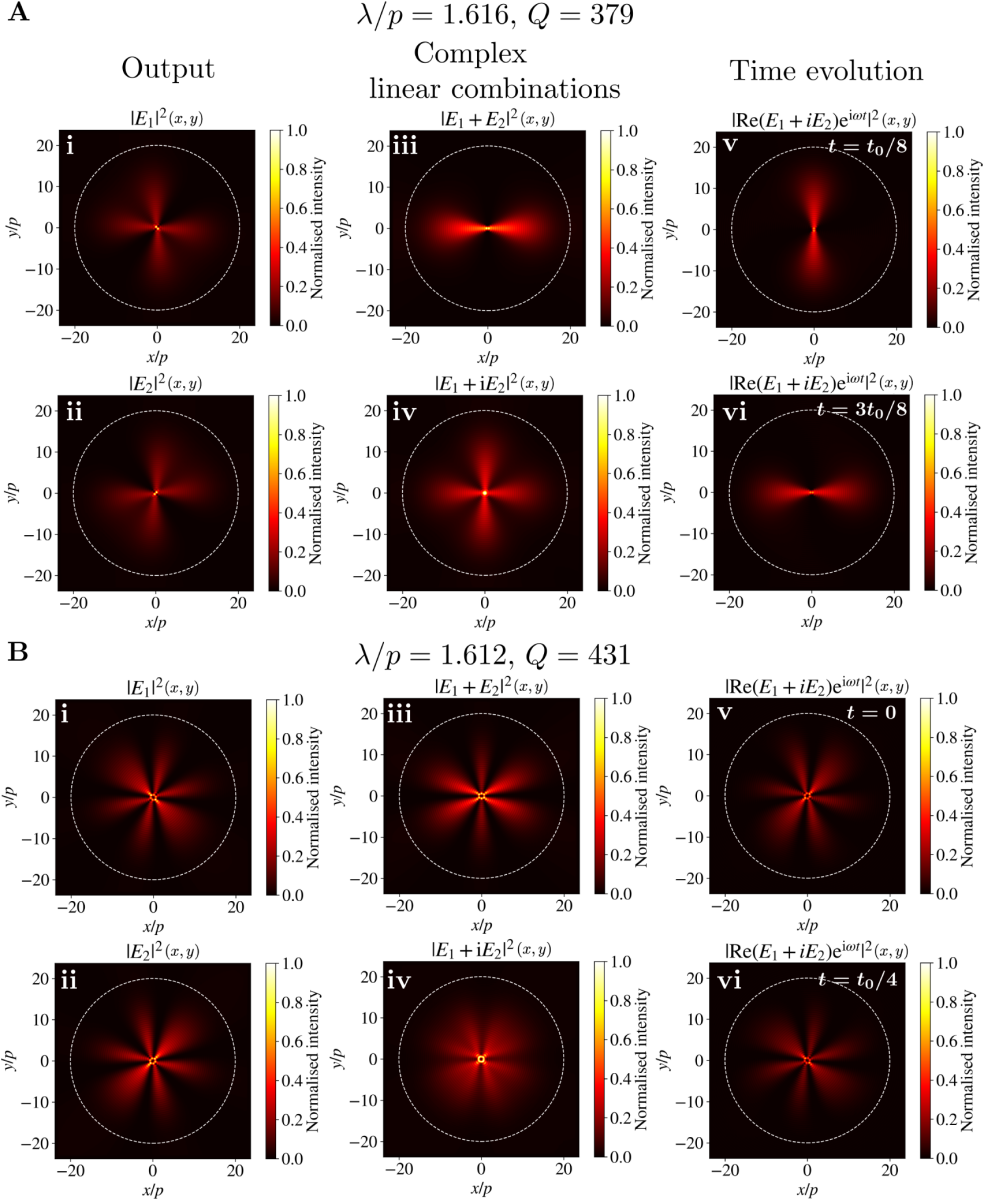
Degenerate modes. Panels (**i**) and (**ii**) show two pairs of degenerate outputs for two selected edge modes (**A**) and (**B**). Panels (**Aiii**) and (**Biii**) show a selected linear combination (a sum) of the output degenerate modes. Panels (**Aiv**) and (**Biv**) show the complex sum, essentially a sum with the phase difference of π, of the output modes. Such complex linear combination represents a traveling wave within the resonator. Panels (**v**) and (**vi**) show the amplitude of the real component of the electric field at different times of the modes shown in panels (**iv**), where *t*_0_ = 2π/ω.

Overall, the edge modes of the 2D cylindrical resonator therefore possess similar features as the edge modes in 1D resonators, i.e., similar frequencies, similar shapes of the electric field envelope, and an approximately constant angle between ***E*** and ***n***. Nevertheless, multiple families of edge modes, recognized as different branches in the spectrum, emerge, due to higher-order resonances occurring in the radial as well as in the azimuthal directions.

### 4.3 Defect Modes

Spectra of 2D cylindrical CLC resonators exhibit a large number of modes in the range of wavelengths that are normally within the band gap range of 1D CLC resonators, i.e., ranging between λ/*p* = *n*_*o*_ and λ/*p* = *n*_*e*_. In 1D CLC systems, modes in band gap can emerge only if a defect is introduced, either in the form of a helix discontinuity (a so-called twist defect, which is shown in Figure 5A) or when an isotropic layer is added between two layers of CLC.^72^ The spectrum of such a 1D CLC resonator with the twist defect is shown in Figure 5B where the defect mode can be recognized as an isolated mode within the band gap. The electric field of the defect modes is localized around the discontinuity as it cannot propagate through the CLC, since its frequency lies in the frequency range of selective light reflection. The main features of the defect modes also include exponential *Q*-factor growth when the thickness of the CLC layers is increased^72^ and exponential field decay in the CLC layer with increased distance from the defect (see, for example, Figure 5C).

**Figure 5:**
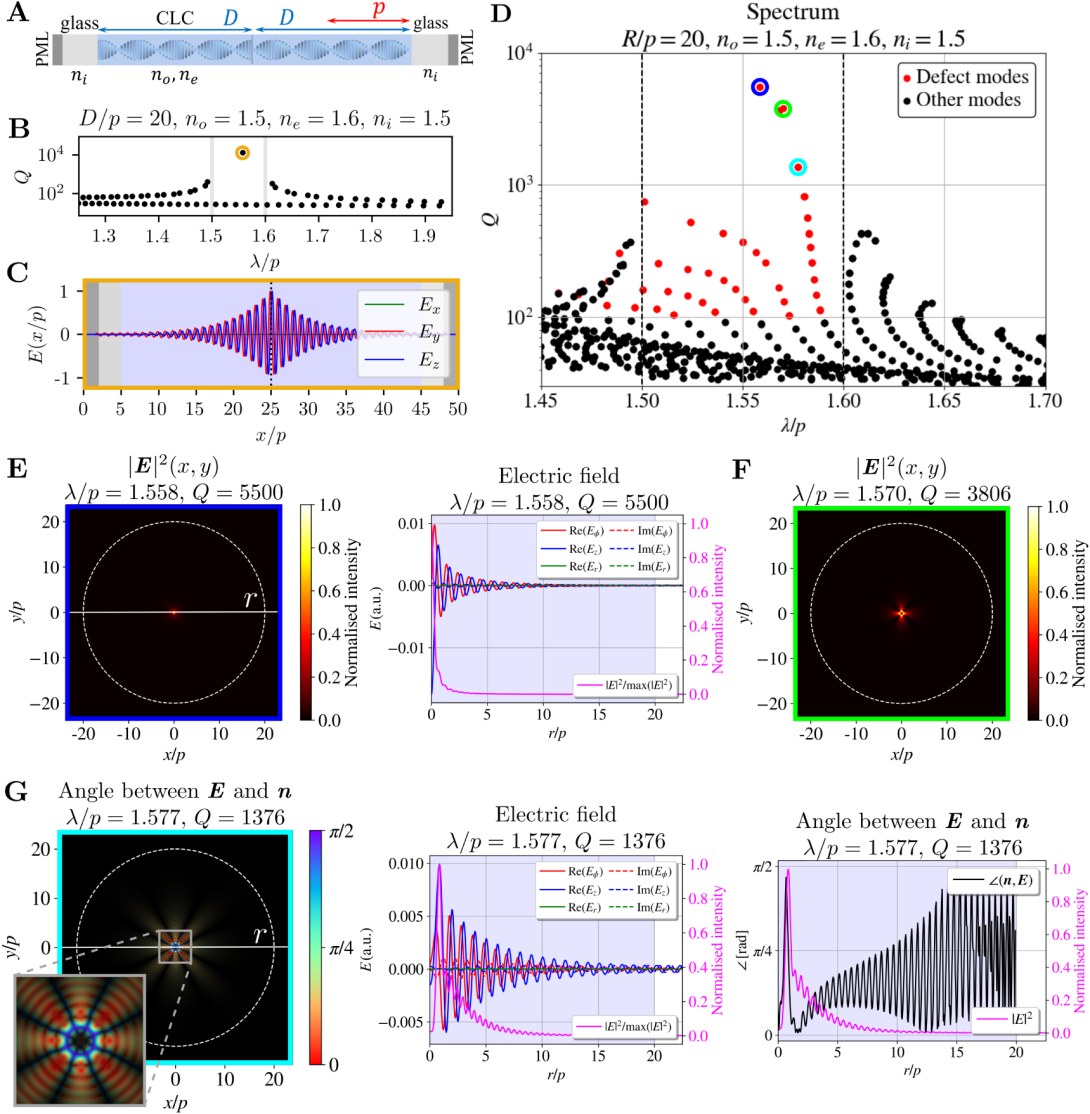
Defect modes in 1D and in 2D CLC cylindrical resonators. (**A**) Schematic of a 1D CLC resonator with a 90° twist defect. (**B**) Spectrum of the resonator with the twist defect. (**C**) Electric field profile of the basic twist defect mode. (**D**) Spectrum of the 2D cylindrical CLC resonator with marked defect modes. (**E**) 2D electric field intensity profile (left) and electric field (red, blue, and green) and electric field intensity (pink) profiles in the radial direction (bottom panel) of the defect mode with the highest *Q*-factor. (**F**) 2D electric field intensity profile of another selected defect mode. (**G**) Left: Color-coded angle between ***E*** and ***n***. Brightness corresponds to the intensity of the mode. Center: same angle in the radial direction at the polar angle of the intensity maximum. Right: electric field and electric field intensity profiles. Positions of all three modes in the spectrum are marked with the colors of the squares around the 2D profiles.

The director field that we use in our analysis of the 2D cylindrical resonator does not include a defect or a discontinuity; however, we still observe defect modes in the calculated spectrum (for *R*/*p* = 20, *n*_*o*_ = 1.5, and *n*_*e*_ = 1.6), as shown in Figure 5D, where we plot defect modes with *Q* > 100 as red dots. Defect modes in the 2D CLC cylindrical resonator are localized around the symmetry point, which is the center of the cylinder, as shown by the electric field intensity profiles in *xy*-plane, which are shown in Figure 5E (left) and Figure 5F. In Figure 2A, we can notice that *Q*-factors of the defect modes increase with larger radii of the 2D cylindrical resonator (see modes within the 1D band gap in Figure 2A). On the other hand, *Q*-factors of the defect modes can drop when the birefringence of the CLC is increased, which we also observe in Figure 2B. Whether *Q*-factors of defect modes will increase or decrease with higher birefringence depends on the radius of the CLC cylinder.

2D electric field, electric field intensity profiles, and the angle between the electric field and the director for selected defect modes are shown in Figure 5E–G. The calculated defect modes in general have constant linear polarization in the center (in *z*-direction, see maxima of blue curves in Figure 5E-right and Figure 5G-center), which then gradually starts to rotate at an approximately constant angle with the director field as we move away from the center. This constant rotation is observed as a constant color in the color-coded plot of an angle between ***E*** and ***n*** (Figure 5G-left) and results in the selective light reflection, which traps the light in the center of the cylinder. Similar to edge modes, also 2D defect modes exhibit an envelope of the standing wave as the 1D defect modes, and their *Q*-factors are of the same order of magnitude. The emergence of multiple families of defect modes is associated with higher orders appearing in the azimuthal direction, which are recognized as petal structures in Figure 5F,G.

### 4.4 Hybrid WG-Defect Modes

In addition to the edge modes and defect modes that can be observed also in 1D geometries, another set of modes emerges in the 2D CLC resonator when we increase the birefringence of the CLC, as we can see from the spectra in Figure 6A–D. Specifically, we use *n*_*o*_ = 1.5 and *n*_*e*_ = (1.6, 1.65, 1.7, 1.75). According to the electric field and electric field intensity profiles of these modes (two examples are shown in Figure 6E,F), they possess characteristics of both whispering gallery modes (WGMs) and defect modes. Therefore, we refer to them as hybrid WG-defect modes.

**Figure 6:**
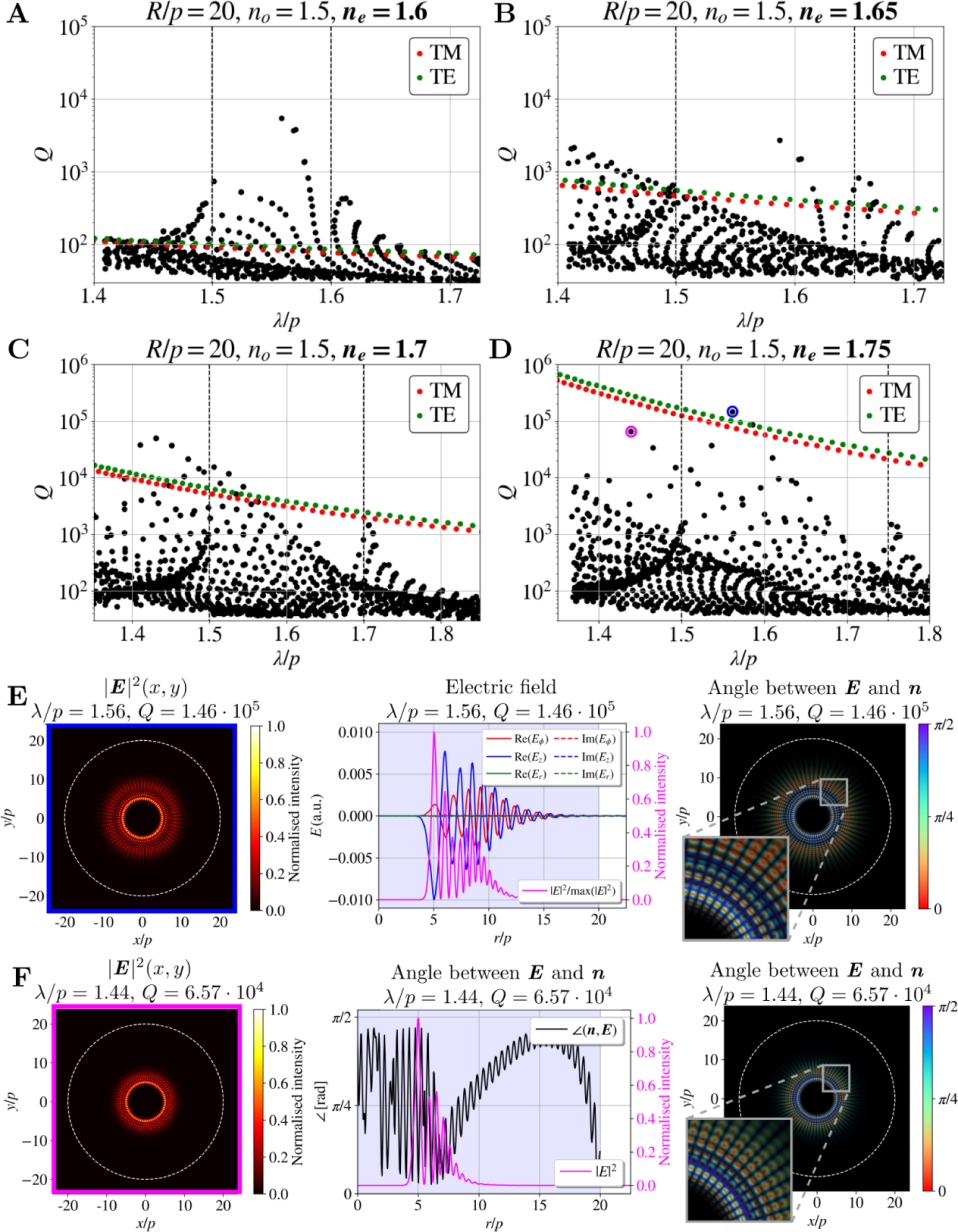
Hybrid WG-defect modes. (**A**–**D**) Spectra of 2D CLC resonator with *R*/*p* = 20, *n*_*o*_ = *n*_*i*_ = 1.5 and (**A**) *n*_*e*_ = 1.6, (**B**) *n*_*e*_ = 1.65, (**C**) *n*_*e*_ = 1.7, and (**D**) *n*_*e*_ = 1.75. Note the different ranges of axes in (**A**,**B**) and (**C**,**D**). Red and green dots mark the calculated whispering gallery modes (WGMs) with TM and TE polarization, respectively, that would emerge at the edge of an isotropic cylindrical or spherical resonator with *R*/*p* = 20, the refractive index *n*_*e*_ in an isotropic environment with the refractive index *n*_*o*_. (**E**,**F**) Two selected examples of hybrid WG-defect modes. Far-left panels show the electric field intensity profile; far-right panels show the angle between the electric field and director. The middle panel in (**E**) shows the electric field profile in the radial direction, while the middle panel in (**F**) shows the angle between the electric field and director in the radial direction.

Whispering gallery modes^73,74^ are a type of optical resonance that occurs when light waves are confined within a circular or spherical structure, such as a cylinder, droplet, or microdisk. In these structures, light waves travel along the perimeter and form a standing wave resonance via total internal reflection, which requires a refractive index gradient, typically occurring as a boundary between two materials with different refractive indices. Differently, in CLC the refractive index gradient for a certain polarization occurs due to the layered CLC structure. CLC layers with high enough birefringence could potentially support WGMs within the bulk, but typically, the continuous nature of the CLC structure with the gradual reorientation of the director field results in insufficient reflectivity to sustain WGMs with similar *Q*-factors to WGMs that would occur at the cylindrical boundary between isotropic media (red and green dots in Figure 6A–D). The reflectance on specific layers is effectively enhanced by selective light reflection within the bulk CLC. Consequently, hybrid modes are formed, consisting of a WGM component at a specific layer in the CLC cylindrical structure due to total internal reflection and a defect-like exponentially decaying component due to selective light reflection.

This hybrid structure can be observed in the intensity plots and the angle between the electric field and the director field in Figure 6E,F. Initially, at smaller radii, a WGM intensity peak appears as a bluish region with the electric field polarized in the *z*-direction and not following the rotation of the director field. At larger radii, an intensity dip occurs, followed by the electric field aligning with the director field’s rotation, enhancing the selective light reflection, similar as in the defect modes. Later can be seen by comparing the insets in Figures 5G and 6E,F.

All observed hybrid modes exhibit a similar structure, differing mainly in the radii where the WGM component occurs. Hybrid WG-defect modes have the highest *Q*-factors near the blue edge of the band gap, as lower wavelengths enhance WGMs’ total internal reflection (see red and green dots in Figure 6A–D), while defect modes achieve peak *Q*-factors near the center of the band gap. The specific frequency and *Q*-factor values depend on a complex interplay of the parameters. For instance, a smaller WGM radius may reduce the *Q*-factor due to weaker internal reflections but would strengthen selective reflection because of the thicker surrounding CLC layer. Consequently, multiple families of hybrid WG-defect modes with varying WGM radii and contributions from total internal and selective light reflection are observed in the spectrum. It is important to note that for the explored range of birefringence values, the edge modes on the red side of the band gap remain relatively isolated from the hybrid WG-defect modes (see the cluster of modes near the right vertical dashed line). This isolation makes them promising candidates for lasing applications.

### 4.5 Boundary Whispering Gallery Modes

WGMs at the boundary between the resonator and the surrounding are observed if the refractive index of the isotropic surrounding is sufficiently small, compared to the refractive indices of the CLC. Spectra of the 2D CLC resonator with *R*/*p* = 20, *n*_*o*_ = 1.5, *n*_*e*_ = 1.7, surrounded by isotropic media with refractive indices *n*_*i*_ = 1.33 and *n*_*i*_ = 1, are shown in Figure 7A,B, representing a CLC resonator in water or air, respectively. Both panels also include the spectrum for a resonator with a matched surrounding refractive index of *n*_*i*_ = 1.5. In Figure 7A, 2 families of WGMs are evident. The family with higher *Q*-factors consists of first radial order WGMs, characterized by a single intensity maximum in the radial direction. An example of such a first radial order WGM is shown in Figure 7C, with the 2D electric field intensity profile on the left and the radial electric field and electric field intensity intensity (pink) profiles on the right, demonstrating characteristic field localization at the boundary. Second radial order WGMs are also observed, with *Q*-factors about 2 orders of magnitude lower. An example of a second radial order WGM is shown in Figure 7D, where the color code represents the angle between the electric field and the director, and the brightness corresponds to the electric field intensity. The inset shows how the angle changes rapidly in the radial direction, as the director field rotates in a helical fashion, while the electric field preserves constant polarization in the *z*-direction.

**Figure 7:**
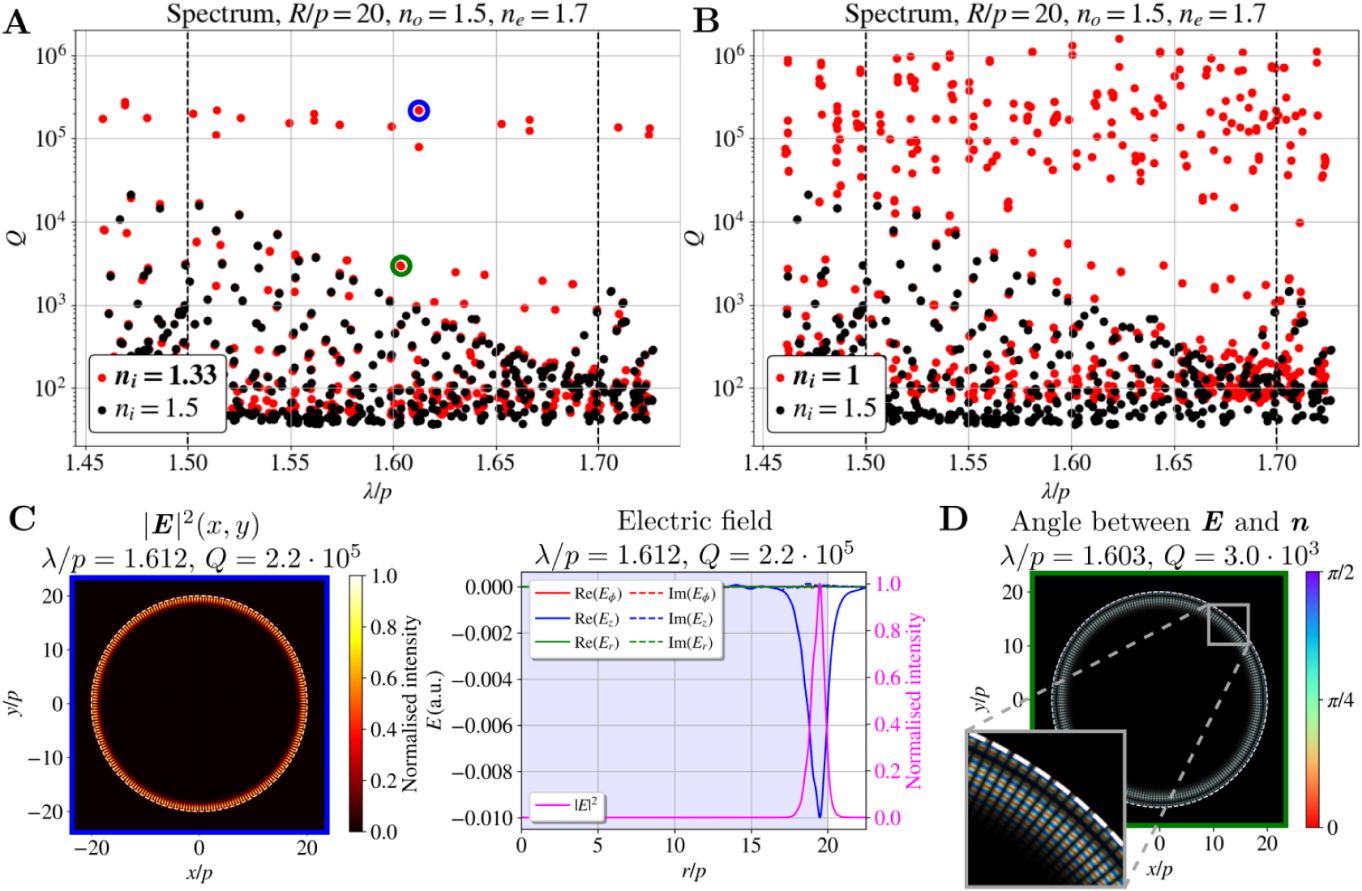
Boundary whispering gallery modes. Spectra of the 2D CLC resonators with *R*/*p* = 20, *n*_*o*_ = 1.5, *n*_*e*_ = 1.7, and refractive index of the isotropic surrounding (**A**) *n*_*i*_ = 1.33 and (**B**) *n*_*i*_ = 1 are plotted with red dots. For reference, the spectrum of the same resonator with an ordinary index-matched outside layer *n*_*i*_ = 1.5 is plotted with black dots in both panels. (**C**) 2D electric field intensity profile (left) and electric field (red, blue, and green) and electric field intensity (pink) profiles in the radial direction (right) of a selected first radial order WGM. (**D**) Angle between electric field ***E*** and director ***n***. Brightness corresponds to the electric field intensity | ***E*** |^2^.

A significantly larger number of WGMs is observed when *n*_*i*_ = 1 is used (see Figure 7B) because the third and higher radial order WGMs are also present in this case. Different radial order WGMs are not well distinguished from each other in terms of *Q*-factors, likely due to the limitations imposed by the resolution of the square mesh used in the calculations. Importantly, the radius at which intensity maxima occur in the observed WGMs of the highest radial order is still significantly larger than the radius at which intensity maxima occur in hybrid WG-defect modes, making these two types of modes easily distinguishable from each other. It is noteworthy that WGMs also occur when the refractive index of the matrix is matched with the ordinary refractive index of LC (*n*_*i*_ = *n*_*o*_, as in previous sections) for higher values of birefringence (*n*_*e*_ = 1.8). However, their *Q*-factors are one to 2 orders of magnitude smaller than the *Q*-factors of the hybrid WG-defect modes and are therefore not clearly distinguished in the spectrum.

### 4.6 Photonic Modes of Spiral CLC Structure—Toward Understanding Photonic Modes of 3D CLC Droplets

We explore another CLC profile—i.e., the birefringence—that can possibly emerge in two-dimensional CLC resonators, which is characterized by a spiral director profile (see Figure 8A). Actually, note that this profile can be seen as a *cross-section* within a typical structure of a CLC spherical droplet (also known as the RSS structure,^66,71^ as shown in Figure 1D,E). We write the spiral profile by using the analytical formula:^71^

9 $$n\left(r,\phi \right)=\left(n_{x}\left(r,\phi \right),n_{y}\left(r,\phi \right),n_{z}\left(r,\phi \right)\right)=\left(-\sin\Theta \;\sin\phi ,\sin\Theta \;\cos\phi ,\cos\Theta \right)$$

where

10 $$\Theta =\phi +\frac{2\pi r}{p}$$

and $r=\sqrt{x^{2}+y^{2}}$ and ϕ = atan2­(*y*, *x*) are cylindrical coordinates. The spectra of the 2D CLC resonator with a spiral structure and *R*/*p* = 20, *n*_*o*_ = 1.5, *n*_*i*_ = 1.5 are shown in Figure 8A for *n*_*e*_ = 1.6 and Figure 8B for *n*_*e*_ = 1.7. As the director profile has a discontinuity in the center (*r* = 0), we have assumed that the birefringence of the profile changes from Δ*n* to 0 in the central area with *r* < *p*/4. Such an approach approximates the presence of a point defect in the center.

**Figure 8:**
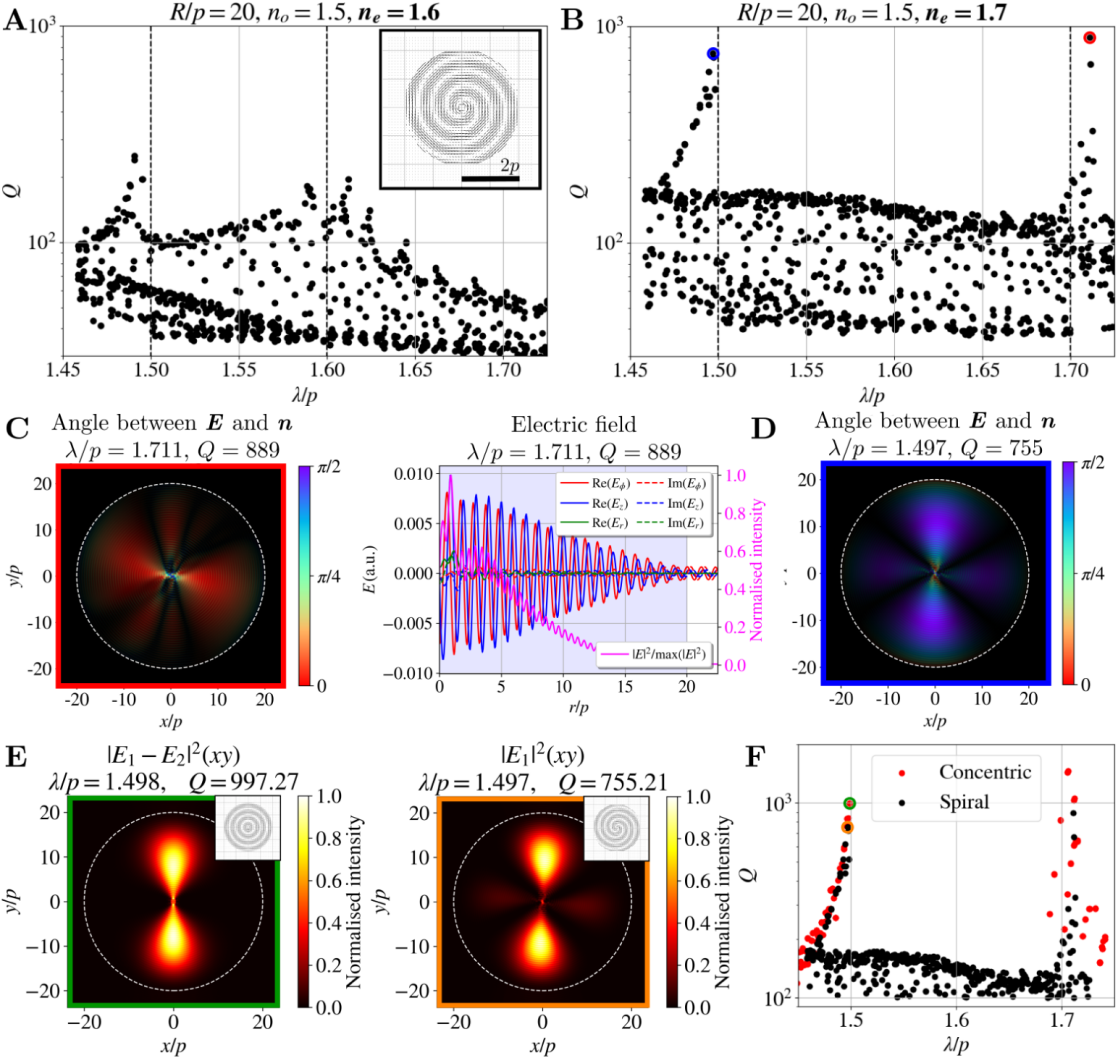
Spectrum of the CLC cylindrical profile with a spiral cross section. Spectra are shown for *R*/*p* = 20, *n*_*o*_ = 1.5, *n*_*i*_ = 1.5, and (**A**) *n*_*e*_ = 1.6, (**B**) *n*_*e*_ = 1.7. The inset in (**A**) shows the director field of the spiral cross section for *R*/*p* = 2. (**C**) Edge mode with the highest *Q*-factor. The left panel shows the angle between the electric field ***E*** and the director ***n*** (color code). Brightness corresponds to the electric field intensity | ***E*** |^2^. The right panel shows the electric field and electric field intensity in the radial direction at a polar angle corresponding to the intensity maximum. (**D**) Angle between ***E*** and the director ***n*** (color code) and intensity (brightness) of the mode with the second highest *Q*-factor. (**E**) Comparison of equivalent edge modes in radial concentric and spiral structures. The mode of the concentric structure is obtained as a linear combination of two degenerate modes. (**F**) Comparison of the spectrum of the spiral structure with the edge modes of the radial structure.

Since the spiral structure is not circularly symmetric, it does not support WGM resonances, as the CLC layers do not form circles with fixed perimeters but a spiral. Therefore, the possible total internal reflection at the layers does not lead toward the standing wave, but guides the light toward the boundary of the droplet. However, despite the spiral structure, the edge modes still occur and are, contrary to the layered structure, clearly seen in the spectrum at the edges of the 1D band gap. Edge modes with the highest *Q*-factors on the red (i.e., longer wavelength) and the blue (i.e., shorter wavelength) edge are shown in Figure 8C,D, respectively. A characteristic alignment of the electric field with the nematic director field is observed. Due to the lack of symmetry, *Q*-factors of edge modes in the spiral structure are smaller than the *Q*-factors of edge modes in the layered structure with the same birefringence. A direct comparison between equivalent modes for the spiral and concentric structure with *n*_*e*_ = 1.7 is shown in Figure 8E. The comparison between the spectrum of the edge modes of the concentric structure and the spectrum of the spiral structure is shown in Figure 8F and shows that the frequencies of the modes remain roughly the same, but the *Q*-factors are slightly decreased in the spiral structure. The edge modes of the concentric structure are identified by using the following criterion for the intensity-weighted average angle between ***E*** and ***n***, as defined by eq 8: |∠(***n***, ***E***)| < 30° or |∠(***n***, ***E***)| > 60°.

Understanding the modes of the 2D cross sections of the RSS birefringent structure (as shown in Figure 1C–E) can provide valuable insights into the optical modes of 3D spherical cholesteric droplets. This is particularly relevant since analyzing the 3D problem directly is highly challenging due to high computational memory requirements (the number of eigenmatrix elements scales as *R* ^6^ in 3D). Consequently, only droplets with very small radii can be analyzed within a reasonable time frame by using current computational resources. Although the modes of 3D spherical CLC resonators may differ from those presented here, due to the presence of escaped disclination line defects and spherical symmetry, they are likely to exhibit some similar properties. For instance, edge modes, which are often utilized in CLC lasing, are still expected to occur at the edges of the 1D photonic band gap. For the explored parameters and a radius of *R*/*p* = 20, the refractive indices *n*_*o*_ = 1.5 and *n*_*e*_ = 1.7 appear to be the appropriate selection, as edge modes are prominent in both cross sections, and hybrid WG-defect modes do not emerge in their immediate vicinity. Overall, we propose that the most efficient approach to analyzing large 3D systems would involve combining FDFD and FDTD methods, as detailed in the Supporting Information.

## 5 Discussion

This study presents a systematic method for the identification of optical modes in various cholesteric liquid crystal (CLC) geometries, explores the complex optical behavior of 2D CLC cylindrical resonators, including in relation to the much better-known 1D CLC resonators, and can also be applied toward the understanding of optical modes in 3D CLC droplets. By using the Finite Difference Frequency Domain (FDFD) method, we identify Bragg-like edge modes, defect modes, and whispering gallery modes and introduce hybrid WG-defect modes in 2D cylindrical CLC resonators with selected birefringent profiles. The results highlight the significant influence of cylindrical symmetry on optical modes in CLCs, offering new insights into resonance phenomena. Combining cylindrical symmetry with the helical nature of CLC, for example, leads to an interplay between WGM resonances and Bragg reflections, leading to the emergence of a new type of hybrid WG-defect modes. Further analysis is required to fully understand the optical modes in 3D CLC droplets, as these modes may differ from the ones presented here due to the presence of escaped disclination line defects or spherical symmetry. Nevertheless, some control over which modes will occur can likely be achieved by an appropriate selection of the parameters, as presented in this work.

## Supplementary Material











## Data Availability

The data that support the findings of this article are openly available.^75^

## Notes

A comparison of results obtained by Finite Difference Frequency Domain (FDFD) and Finite Difference Time Domain (FDTD) methods is given (PDF)
Investigation on how discretizing a circular domain on a rectangular grid affects the polar symmetry of the eigenvalue problem and mode degeneracy (PDF)
Investigation on how discretizing a square domain on a rectangular grid affects the mode degeneracy (PDF)
Animation of time evolution of output modes and their complex linear combinations which are shown in Figure 4A of the main text (GIF)
Animation of time evolution of output modes and their complex linear combinations which are shown in Figure 4B of the main text (GIF)
