# Supplementary material for: Photonic Eigenmodes of 2D Cylindrical Cholesteric Liquid Crystal Resonators
Source: ACS Photonics. 2025 Sep 26;12(10):5572–85. doi: 10.1021/acsphotonics.5c01294 (PMC12532369; doi:10.1021/acsphotonics.5c01294)
Supplement: Supplementary file 1 [file ph5c01294_si_001.pdf]

Supplementary Information: Photonic eigenmodes of 2D cylindrical cholesteric liquid crystal resonators

Urban Mur,* Jaka Zaplotnik, Martin Horvat, Igor Muševič, and Miha Ravnik

E-mail: urban.mur@fmf.uni-lj.si

1 FDFD vs. FDTD

Here we compare the results of Finite Difference Frequency Domain (FDFD) calculations, as presented in the main text, with those obtained using the more established Finite Difference Time Domain (FDTD) method for the same system. This comparison is necessary because the FDFD method becomes impractical for large, possibly 3D structures, primarily due to high memory requirements. The memory constraint can be bypassed by using the FDTD method. To calculate the resonance modes with FDTD, a narrow bandwidth excitation pulse is introduced into the system, and the structure's response is analyzed using a harmonic inversion algorithm of the signal, probed in a selected position. For the analysis, we use the HARMINV function in the Meep open-source FDTD simulation package.[1] The aim is to determine whether a combination of FDFD and FDTD analyses could enhance our understanding of optical resonances in larger systems.

In Figure 1, we present the spectrum and electric field intensity profiles of two selected eigenmodes, calculated using both the FDFD (Figure 1A,C) and FDTD (Figure 1B,D) methods. The modes obtained via FDTD, for a selected central frequency of the excitation pulse

and a specific probe position, are represented by transparent black dots. To obtain the spectrum shown in Figure 1B, 200 excitation pulses with different central frequencies, corresponding to λ/p = 1.45-1.65, were used for each of the eight different positions of the signal probe. Notably, only a limited number of modes were identified in each run of the FDTD method, as the modes excited and detected depend significantly on the excitation pulse frequency and the probe position.

The FDTD spectrum (Figure 1B) demonstrates that a single run of the FDTD method does not provide reliable results. For instance, the light grey areas near the positions where the eigenmodes are expected, according to the FDFD, indicate that many of the detected modes are not true eigenmodes of the system. These solutions are likely superpositions of modes, which could not be resolved due to the proximity of their wavelengths and/or Q-factors (note that regions of light grey dots in general occur, where multiple high Q-factor modes with similar wavelengths are expected). However, by combining the results from multiple FDTD runs, we can more accurately predict which solutions correspond to the actual eigenmodes. These are clearly observed as dark spots in the spectrum, appearing where solutions with similar wavelengths and Q-factors are consistently found across different simulation runs.

In Figure 1C and 1D, we display two selected eigenmodes at the same position in the spectrum, obtained using the FDFD and FDTD methods, respectively. The FDTD mode profile is a snapshot of the field after the mode is excited with an extremely narrow pulse at the exact frequency of the mode. This highlights another drawback of the FDTD method, as an additional simulation is required to extract the mode profile once the Q-factor and λ/p are already known. The mode profiles and wavelengths of the mode with the highest Q-factor, shown on the left side of both panels, match well between the two methods. The discrepancy in the calculated Q-factors likely arises because high Q-factors are difficult to determine accurately using FDTD. The Q-factor is calculated from the time decay of the modes, and since better resonances take longer to decay, longer simulations would be required

for higher precision. Notably, the modes shown on the right side of both panels are different. We found that the mode identified by FDTD is actually a superposition of two modes detected by FDFD, which have very similar both wavelengths and Q-factors, as shown in Figure 1E (same happens for the blue edge mode, shown in Figure 3H of the main text). It is unclear whether these two modes genuinely exist or if the splitting is a numerical artifact, potentially caused by factors such as the finite square mesh or simply numerical precision. In any case, modes that are so close together in terms of both Q-factor and λ/p are likely to be excited simultaneously in practice, as observed in the FDTD results.

Overall, either method can be used to extract the eigenmodes of a given system. Calculations using FDFD are more straightforward, but may require significant memory for large systems. This limitation can be mitigated by employing the FDTD method, although multiple long simulations will be necessary to achieve reliable results. We suggest that the most efficient approach for analyzing large 3D systems would be to first analyze a simplified system with similar geometry—such as one with lower resolution or smaller size—using the FDFD method. The results can then serve as an informed guess for the positions and shapes of the modes in the larger system, which can subsequently be refined using FDTD methods. Together, FDFD and FDTD could provide a powerful toolkit for exploring the optical properties of CLC resonators, facilitating advancements in photonic devices like tunable lasers and sensors.

References

(1) Oskooi, A. F.; Roundy, D.; Ibanescu, M.; Bermel, P.; Joannopoulos, J. D.; Johnson, S. G. MEEP: A flexible free-software package for electromagnetic simulations by the FDTD method. *Computer Physics Communications* **2010**, *181*, 687–702.

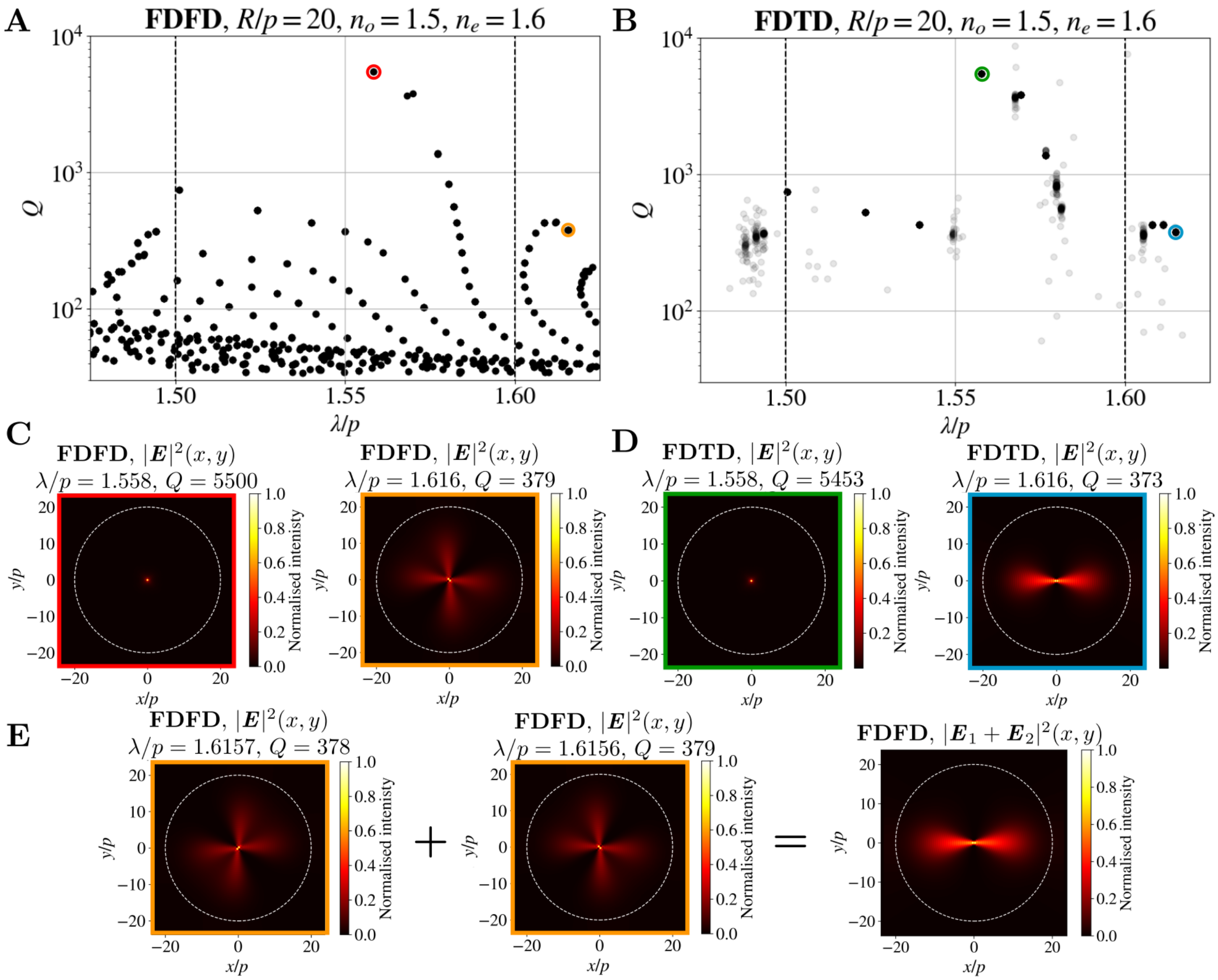

Figure 1: Comparison of results obtained by FDFD and FDTD method. (**A**) Spectrum calculated by FDFD method. (**B**) Spectrum calculated by FDTD method. Each point plotted as a transparent black dot represents a result obtained for a selected central frequency of the input excitation pulse and a selected position of the signal detector. (**C**) Two selected eigenmodes calculated by FDFD method. (**D**) Eigenmodes with similar wavelengths and Q-factors calculated by FDTD method. (**E**) The edge mode obtained by the FDTD method is a sum of two nearly degenerate modes obtained by the FDFD method.
