# Supplementary material for: Photonic Eigenmodes of 2D Cylindrical Cholesteric Liquid Crystal Resonators
Source: ACS Photonics. 2025 Sep 26;12(10):5572–85. doi: 10.1021/acsphotonics.5c01294 (PMC12532369; doi:10.1021/acsphotonics.5c01294)
Supplement: Supplementary file 2 [file ph5c01294_si_002.pdf]

drum_helmholtz

September 1, 2025

1 Helmholtz equation on unit-drum – grid disrupting symmetry

We investigate how discretizing a circular domain on a rectangular grid affects the polar symmetry of the eigenvalue problem. As a case study, we consider the spectrum of a unit disk ('drum') governed by the Helmholtz equation with Dirichlet boundary conditions.

Author: Martin Horvat, August 2025

Ref:

- https://en.wikipedia.org/wiki/Helmholtz_equation
- https://math.libretexts.org/Bookshelves/Differential_Equations/Introduction_to_Partial_Differential_Eq

1.1 Spectrum of the unit-radius drum (Dirichlet Helmholtz problem).

Problem. On the unit disk $D = \{(r, \theta) : 0 \le r < 1, 0 \le \theta < 2\pi\}$ with Dirichlet boundary $u|_{r=1} = 0$, the Helmholtz eigenproblem is

$$\begin{cases} \nabla^2 u + \lambda u = 0, & r < 1, \\ u(1, \theta) = 0. \end{cases}$$

Exact spectrum (closed form). Let J_m be the Bessel function of the first kind of order m. Denote by $j_{m,n}$ the n-th positive zero of J_m. Then the Dirichlet eigenvalues are

$$\boxed{\lambda_{m,n} = j_{m,n}^2, \qquad m = 0, 1, 2, \dots, \; n = 1, 2, \dots}$$

with corresponding eigenfunctions (in polar coordinates)

$$u_{m,n}(r, \theta) = J_m(j_{m,n} r) \times \begin{cases} 1 & (m = 0) \\ \cos(m\theta) \text{ or } \sin(m\theta) & (m \ge 1), \end{cases}$$

so each $m \ge 1$ eigenvalue has **multiplicity** 2 **(cosine & sine)**, while $m = 0$ modes are simple.

1.2 First few eigenvalues (numerical)

Below are the first nine eigenvalues ordered by increasing value (unit-radius disk). These use the standard numerical values of Bessel zeros.

index	(m,n)	zero $j_{m,n}$	eigenvalue $\lambda_{m,n} = j_{m,n}^2$
1	(0,1)	2.4048255576957724	5.783185962946783
2	(1,1)	3.8317059702075125	14.681970642123895
3	(2,1)	5.135622301840683	26.374616427163392
4	(0,2)	5.520078110286311	30.471262343662087
5	(1,2)	7.015586669815619	49.218456321694600
6	(2,2)	8.417244140399866	70.849998919095880
7	(0,3)	8.653727912911013	74.887006790695180
8	(1,3)	10.173468135062722	103.499453895136570
9	(2,3)	11.619841172149060	135.020708865970450

1.3 Numerics

[ ]:

```
import numpy as np
from scipy.sparse import lil_matrix
from scipy.sparse.linalg import eigsh
import matplotlib.pyplot as plt
import numpy as np
from scipy.special import jn_zeros

def drum_spectrum_analytic(m_order, n_display, mult = False):
    """
        Computing first n_display eigenvalues using first m_order.
    """

    rows = []
    for m in range(m_order):
        zeros = jn_zeros(m, n_display)
        for n, z in enumerate(zeros, start=1):
            rows.append((m, n, float(z), float(z*z)))

    # sort by eigenvalue
    spectrum = sorted(rows, key=lambda r: r[3])[:n_display]

    if not mult: return spectrum

    rows = []
    for r in spectrum:
        rows.append(r)
        if r[0] != 0: rows.append(r)

    return rows[:n_display]


def drum_spectrum_fd(N, M):
    """
```

```
    Compute the first M eigenvalues and eigenvectors of the Helmholtz equation
    on a unit disk with Dirichlet boundary conditions using finite differences
    on an N x N grid. Using 5-point stencil which approximates Laplacian␣
↪(nabla^2)
    up to O(h^2).

    Ref:
      * https://www.math.uci.edu/~chenlong/226/FDM.pdf
      * https://en.wikipedia.org/wiki/Five-point_stencil

    Parameters
    ----------
    N : int
        Number of grid points along each axis (domain is [-1, 1] x [-1, 1]).
    M : int
        Number of smallest eigenvalues/eigenvectors to compute.

    Returns
    -------
    result : dict
        {
            "eigenvalues": ndarray of shape (M,),
            "eigenvectors": ndarray of shape (num_points, M),
            "points": ndarray of shape (num_points, 2),
            "X": 2D array (N x N),
            "Y": 2D array (N x N),
            "mask": 2D boolean array (N x N),
            "idx_map": 2D int array (N x N),
            "grid_spacing": float
        }
    """
    # Create grid on [-1, 1] x [-1, 1]
    x = np.linspace(-1, 1, N)
    y = np.linspace(-1, 1, N)
    h = x[1] - x[0]
    X, Y = np.meshgrid(x, y, indexing='ij')

    # Points inside the unit disk
    mask = X**2 + Y**2 <= 1.0

    # Map interior points to 1D indices
    idx_map = -np.ones((N, N), dtype=int)
    idx_map[mask] = np.arange(np.sum(mask))
    num_points = np.sum(mask)

    # Build sparse Laplacian using 5-point stencil
    L = lil_matrix((num_points, num_points))
```

```
    for i in range(N):
        for j in range(N):
            if not mask[i, j]:
                continue
            row = idx_map[i, j]
            L[row, row] = -4.0
            for di, dj in [(-1,0), (1,0), (0,-1), (0,1)]:
                ni, nj = i + di, j + dj
                if 0 <= ni < N and 0 <= nj < N and mask[ni, nj]:
                    col = idx_map[ni, nj]
                    L[row, col] = 1.0

    # Scale and convert to CSR
    L = L.tocsr() / (h**2)

    # Compute M smallest eigenpairs
    vals, vecs = eigsh(L, k=M, which='SM')
    vals = -vals  # negate to match Helmholtz eigenvalues

    # Sort by eigenvalue
    order = np.argsort(vals)
    vals, vecs = vals[order], vecs[:, order]

    # Collect outputs in a dictionary
    return {
        "eigenvalues": vals,
        "eigenvectors": vecs,
        "points": np.vstack((X[mask], Y[mask])).T,
        "X": X,
        "Y": Y,
        "mask": mask,
        "idx_map": idx_map,
        "grid_spacing": h
    }

def plot_drum_eigenfunctions(result, num_modes=6, cmap='viridis', indices =␣
 ↪None):
    """
    Plot the first `num_modes` eigenfunctions of the drum using the output of␣
 ↪drum_spectrum_fd.

    Parameters
    ----------
    result : dict
        Dictionary returned by drum_spectrum_fd.
    num_modes : int
```

```
        Number of eigenfunctions to plot (starting from the smallest␣
↪eigenvalue).
    cmap : str
        Matplotlib colormap for the plots.
    """
    X, Y = result["X"], result["Y"]
    mask = result["mask"]
    vals = result["eigenvalues"]
    vecs = result["eigenvectors"]

    # Limit number of modes if requested more than available
    num_modes = min(num_modes, vecs.shape[1])

    # Prepare figure
    rows = int(np.ceil(num_modes / 3))
    fig, axes = plt.subplots(rows, 3, figsize=(12, 4 * rows))
    axes = axes.ravel()

    for k in range(num_modes):
        # Reconstruct eigenfunction on full grid
        Z = np.full_like(X, np.nan, dtype=float)  # NaN outside disk
        Z[mask] = vecs[:, k]

        im = axes[k].imshow(Z.T, origin='lower', extent=(-1, 1, -1, 1),␣
↪cmap=cmap)
        if indices is None:
            axes[k].set_title(f"Mode {k+1}\n  = {vals[k]:.4f}")
        else:
            axes[k].set_title(f"Mode {k+1}, Index {indices[k]}\n  = {vals[k]:.
↪4f}")

        axes[k].set_xticks([]); axes[k].set_yticks([])
        fig.colorbar(im, ax=axes[k], fraction=0.046, pad=0.04)

    # Hide unused axes if any
    for ax in axes[num_modes:]:
        ax.axis('off')

    plt.tight_layout()
    plt.show()
```

[2]:
```
# Compute  analytics spectrum
spectrum_sorted = drum_spectrum_analytic(12, 12)

# print table
print("{:>5} {:>6} {:>16} {:>20}".format("idx","(m,n)","j_{m,n}","lambda =␣
↪j^2"))
```

```
for i, (m,n,z,lam) in enumerate(spectrum_sorted, start=1):
    print(f"{i:5d}  ({m},{n})  {z:16.12f}  {lam:20.12f}")
```

```
  idx   (m,n)          j_{m,n}         lambda = j^2
    1   (0,1)    2.404825557696         5.783185962947
    2   (1,1)    3.831705970208        14.681970642124
    3   (2,1)    5.135622301841        26.374616427163
    4   (0,2)    5.520078110286        30.471262343662
    5   (3,1)    6.380161895924        40.706465818200
    6   (1,2)    7.015586669816        49.218456321695
    7   (4,1)    7.588342434504        57.582940903291
    8   (2,2)    8.417244140400        70.849998919096
    9   (0,3)    8.653727912911        74.887006790695
   10   (5,1)    8.771483815960        76.938928333647
   11   (3,2)    9.761023129982        95.277572544037
   12   (6,1)    9.936109524218        98.726272477249
```

[3]:
```
# Compute numeric spectrum for various grid sizes
result50 = drum_spectrum_fd(N=50, M=12)
result51 = drum_spectrum_fd(N=51, M=12)
result100 = drum_spectrum_fd(N=100, M=12)
result101 = drum_spectrum_fd(N=101, M=12)

np.array([result51["eigenvalues"],
          result50["eigenvalues"],
          result100["eigenvalues"],
          result101["eigenvalues"]]).T
```

[3]:
```
array([[ 5.63617687,  5.63367182,  5.71818035,  5.7081116 ],
       [14.29547355, 14.28883225, 14.51356001, 14.48809456],
       [14.2957952 , 14.28883225, 14.51356001, 14.48811363],
       [25.60933499, 25.59277142, 26.03696031, 26.008105  ],
       [25.69075154, 25.68034089, 26.09126026, 26.02918306],
       [29.62084521, 29.60518562, 30.10931524, 30.0568415 ],
       [39.52943041, 39.50595401, 40.21170808, 40.14235139],
       [39.5299362 , 39.50595401, 40.21170808, 40.1423798 ],
       [47.75262405, 47.72422309, 48.61025712, 48.52595336],
       [47.75356294, 47.72422309, 48.61025712, 48.52600366],
       [55.79254026, 55.46137523, 56.71818409, 56.67208379],
       [55.85142096, 56.1154331 , 56.9990347 , 56.84976574]])
```

[21]:
```
# Compute numeric spectrum
result = drum_spectrum_fd(N=100, M=12)

# Print eigenvalues
print("Eigenvalues:", result["eigenvalues"])

# Plot first 12 modes
```

```
indices = [(n,m) for n,m,_,_ in drum_spectrum_analytic(12, 12, mult=True)]
plot_drum_eigenfunctions(result, num_modes=12, indices = indices)
```

```
Eigenvalues: [ 5.71818035 14.51356001 14.51356001 26.03696031 26.09126026
30.10931524
 40.21170808 40.21170808 48.61025712 48.61025712 56.71818409 56.9990347 ]
```

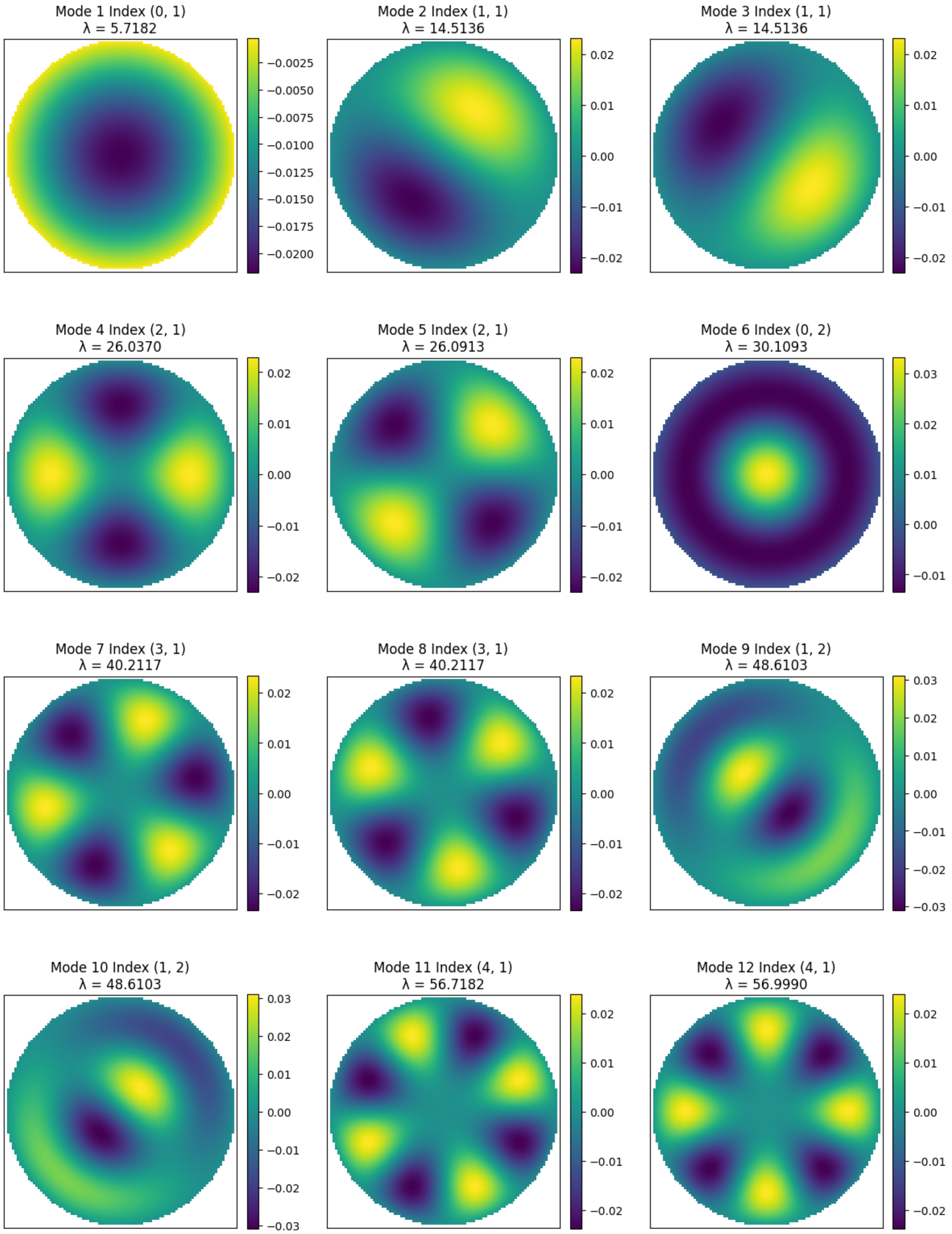

[5]:
```
# Number of first eigenvalues that we want to studz
M_evals = 12

# Compute analytical spectrum of M_evals eigenvalues
sp_ana = drum_spectrum_analytic(M_evals, M_evals, mult = True)
sp_ana_vals = np.array([r[-1] for r in sp_ana])

# Compute relative difference of eigenvalues as the grid is refined
delta_spectrum = []
for N_grid in range(10, 150):
    sp_num_vals = drum_spectrum_fd(N = N_grid, M = M_evals)["eigenvalues"]
    delta_spectrum.append((N_grid, (sp_num_vals - sp_ana_vals)/sp_ana_vals))
```

[6]:
```
# Transform results for easier plotting
res_transf = np.array([[(N_grid, v) for v in vals] for N_grid, vals in␣
 ↪delta_spectrum])
res_transf = np.swapaxes(res_transf, 0, 1)
```

[8]:
```
sp_ana
```

[8]:
```
[(0, 1, 2.4048255576957724, 5.783185962946783),
 (1, 1, 3.8317059702075125, 14.681970642123895),
 (1, 1, 3.8317059702075125, 14.681970642123895),
 (2, 1, 5.135622301840683, 26.374616427163392),
 (2, 1, 5.135622301840683, 26.374616427163392),
 (0, 2, 5.520078110286311, 30.471262343662087),
 (3, 1, 6.380161895923983, 40.706465818200314),
 (3, 1, 6.380161895923983, 40.706465818200314),
 (1, 2, 7.015586669815619, 49.2184563216946),
 (1, 2, 7.015586669815619, 49.2184563216946),
 (4, 1, 7.588342434503804, 57.582940903291124),
 (4, 1, 7.588342434503804, 57.582940903291124)]
```

[9]:
```
fig, ax = plt.subplots(figsize = (10, 7))

# Separately plotting for each eigenvalue
for i, r in enumerate(res_transf):
    ax.plot(r[:,0],-r[:,1], "o-", label =␣
 ↪f"(m,n)=({sp_ana[i][0]},{sp_ana[i][1]})", alpha=0.7)

# adding rough dependence
x = np.linspace(10, 200, 100)
ax.plot(x, x**-1, lw = 2, label = r"$N_{grid}^{-1}$")
```

```
ax.set_xlabel(r"$N_{grid}$")
ax.set_ylabel(r"$1 - \lambda_{i, num}/\lambda_{i,ana}$")

ax.set_xscale("log")
ax.set_yscale("log")

ax.set_title("Relative precision of numerical spectrum")

ax.legend(loc='center left', bbox_to_anchor=(1, 0.5))
plt.show()
```

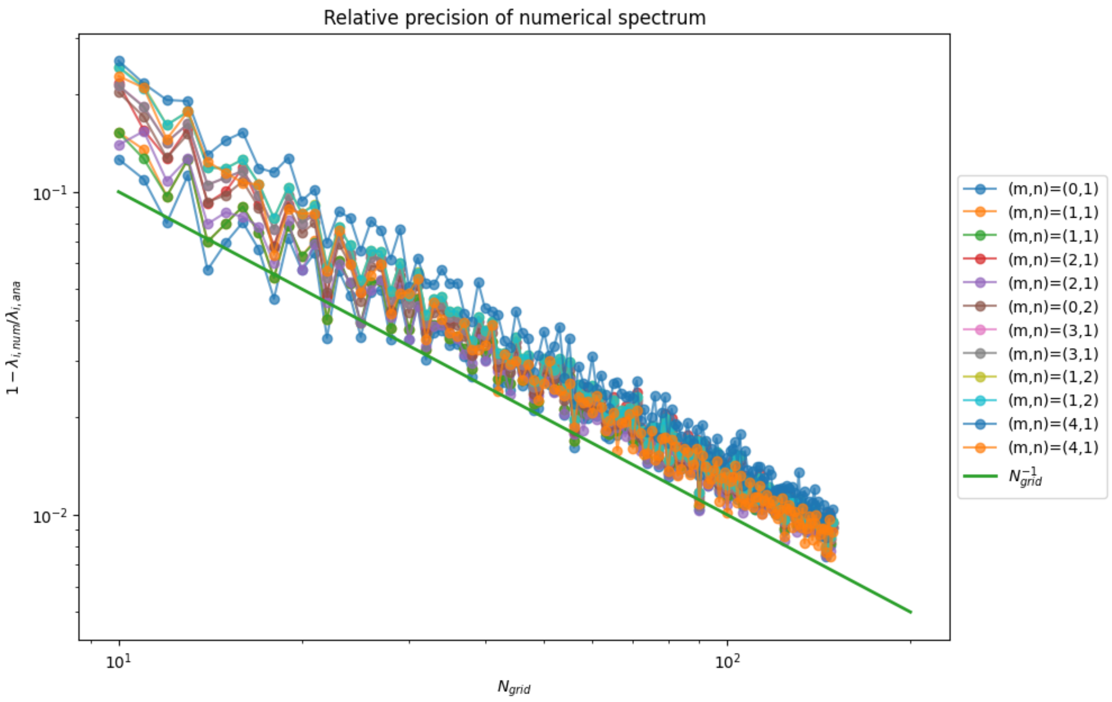

[19]:
```
fig, ax = plt.subplots(figsize = (10, 7))


# Plotting relative difference of near degenerate pairs
idx0 = (None, None)
for i, r in enumerate(res_transf):
    idx = sp_ana[i]

    if (idx[0] == idx0[0]) and (idx[1] == idx0[1]):
        ax.plot(r[:,0], np.abs(v - r[:,1]), "o-", label = f"(m,n) =␣
↪({idx[0]},{idx[1]})", alpha=0.7)
    else:
        v = r[:,1]
```

```
    idx0 = idx[:]

# adding rough dependence
x = np.linspace(10, 200, 100)
ax.plot(x, x**-1, lw = 2, label = r"$N_{grid}^{-1}$")
ax.plot(x, x**-2, lw = 2, label = r"$N_{grid}^{-2}$")
ax.plot(x, x**-3, lw = 2, label = r"$N_{grid}^{-3}$")

ax.set_xlabel(r"$N_{grid}$")
ax.set_ylabel(r"$|(\lambda_{\mathrm{first in pair, num}} -␣
↪\lambda_\mathrm{second in pair, num})|/\lambda_{i,ana}$")

ax.set_title("Relative difference of near degenerate pairs")

ax.set_xscale("log")
ax.set_yscale("log")

ax.legend(loc='center left', bbox_to_anchor=(1, 0.5))
plt.show()
```

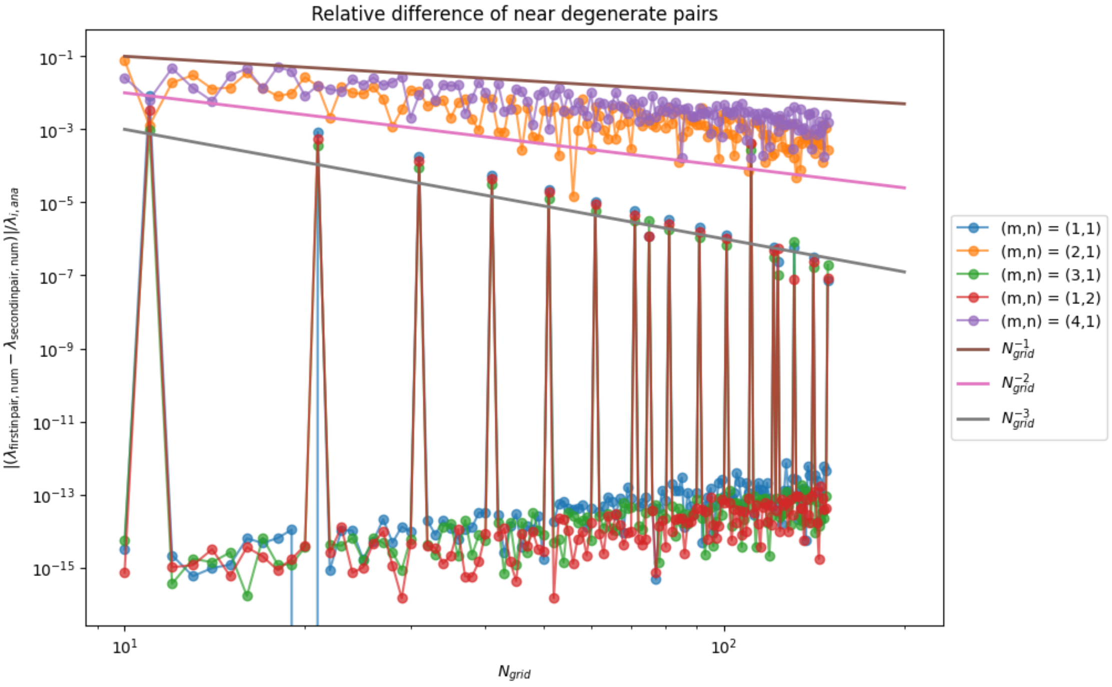

1.3.1 Conclusion

The discrepancy between numerical and exact eigenvalues arises primarily from two sources:

1. **Discretization error** of the finite difference scheme, which scales as $O(h^{-2})$.
2. **Geometric approximation of the domain**, such as representing a circle on a Cartesian grid, introducing an error of order $O(h^{-1})$.

In our case, the geometric error dominates.

The exact spectrum possesses degeneracies of multiplicity 2 for modes with $m > 0$. In the numerical spectrum, these degeneracies are typically broken: instead of exact pairs, we observe nearly degenerate eigenvalues, with the relative difference between paired values scaling as $O(h^{-1})$ in the worst case. But can be smaller. However, if the symmetry of an eigenvalue pair aligns with the symmetry of the grid, the degeneracy can remain highly accurate—sometimes preserved up to unavoidable floating-point round-off error.
