# Supplementary material for: Photonic Eigenmodes of 2D Cylindrical Cholesteric Liquid Crystal Resonators
Source: ACS Photonics. 2025 Sep 26;12(10):5572–85. doi: 10.1021/acsphotonics.5c01294 (PMC12532369; doi:10.1021/acsphotonics.5c01294)
Supplement: Supplementary file 3 [file ph5c01294_si_003.pdf]

square_helmholtz

September 1, 2025

1 Helmholtz equation on unit-square

1.1 Spectrum of the unit square (Dirichlet Helmholtz problem)

Problem On the unit square $\Omega = [0,1] \times [0,1]$ with fixed (Dirichlet) boundary, the Helmholtz eigenproblem is

$$\begin{cases} \nabla^2 u + \lambda u = 0, & (x,y) \in \Omega, \\ u = 0, & (x,y) \in \partial\Omega. \end{cases}$$

Exact spectrum (closed form).

For integers $p, q = 1, 2, 3, \ldots,$

$$\boxed{\lambda_{p,q} = \pi^2 \left(p^2 + q^2\right)}, \qquad \boxed{u_{p,q}(x,y) = \sin(p\pi x)\,\sin(q\pi y)} \text{ (up to normalization).}$$

- Each eigenvalue depends only on $p^2 + q^2$.
- If $p \neq q$, then $\lambda_{p,q} = \lambda_{q,p}$ (multiplicity 2).
- If $p = q$, the eigenvalue is simple (multiplicity 1).

First few eigenvalues (ordered)

index	(p,q)	$\lambda_{p,q}$
1	(1,1)	19.739208802
2	(1,2)	49.348022005
3	(2,1)	49.348022005
4	(2,2)	78.956835209
5	(1,3)	98.696044011
6	(3,1)	98.696044011
7	(2,3)	128.304857214
8	(3,2)	128.304857214
9	(1,4)	167.783274819
10	(4,1)	167.783274819

1.2 Numerics

[10]:
```python
import numpy as np
from scipy.sparse import lil_matrix
from scipy.sparse.linalg import eigsh
import matplotlib.pyplot as plt


def square_spectrum_fd(N, M):
    """
    Compute the first M eigenvalues and eigenvectors of the Helmholtz equation
    on a unit square [0,1]x[0,1] with Dirichlet boundary conditions using
    finite differences on an N x N grid (including boundaries).

    Parameters
    ----------
    N : int
        Number of grid points along each axis (including boundaries).
    M : int
        Number of smallest eigenvalues/eigenvectors to compute.

    Returns
    -------
    result : dict
        {
            "eigenvalues": ndarray of shape (M,),
            "eigenvectors": ndarray of shape (num_points, M),
            "X": 2D array (N x N),
            "Y": 2D array (N x N),
            "grid_spacing": float
        }
    """
    # Grid setup
    x = np.linspace(0, 1, N)
    y = np.linspace(0, 1, N)
    h = x[1] - x[0]
    X, Y = np.meshgrid(x, y, indexing='ij')

    # Interior points (exclude boundaries)
    interior_N = N - 2
    num_points = interior_N * interior_N

    # Build sparse Laplacian for interior points using 5-point stencil
    L = lil_matrix((num_points, num_points))
    for i in range(interior_N):
        for j in range(interior_N):
            row = i * interior_N + j
            L[row, row] = -4.0
```

```
            # Left
            if j > 0:
                L[row, row - 1] = 1.0
            # Right
            if j < interior_N - 1:
                L[row, row + 1] = 1.0
            # Up
            if i > 0:
                L[row, row - interior_N] = 1.0
            # Down
            if i < interior_N - 1:
                L[row, row + interior_N] = 1.0

    # Scale and convert to CSR
    L = L.tocsr() / (h**2)

    # Compute M smallest eigenpairs
    vals, vecs = eigsh(L, k=M, which='SM')
    vals = -vals  # negate for Helmholtz

    # Sort by eigenvalue
    order = np.argsort(vals)
    vals, vecs = vals[order], vecs[:, order]

    return {
        "eigenvalues": vals,
        "eigenvectors": vecs,
        "X": X,
        "Y": Y,
        "interior_N": interior_N,
        "grid_spacing": h
    }


def plot_square_eigenfunctions(result, num_modes=6, cmap='viridis', indices =␣
 ↪None):
    """
    Plot the first `num_modes` eigenfunctions of the unit square using␣
 ↪square_spectrum_fd output.
    """
    vals = result["eigenvalues"]
    vecs = result["eigenvectors"]
    interior_N = result["interior_N"]

    num_modes = min(num_modes, vecs.shape[1])
    rows = int(np.ceil(num_modes / 3))
    fig, axes = plt.subplots(rows, 3, figsize=(12, 4 * rows))
```

```python
    axes = axes.ravel()

    for k in range(num_modes):
        # Reconstruct eigenfunction on full grid (including boundaries)
        Z = np.zeros((interior_N + 2, interior_N + 2))
        Z[1:-1, 1:-1] = vecs[:, k].reshape((interior_N, interior_N))

        im = axes[k].imshow(Z.T, origin='lower', extent=(0, 1, 0, 1), cmap=cmap)

        if indices is None:
            axes[k].set_title(f"Mode {k+1}\n  = {vals[k]:.4f}")
        else:
            axes[k].set_title(f"Mode {k+1}, Index {indices[k]}\n  = {vals[k]:.4f}")

        axes[k].set_xticks([]); axes[k].set_yticks([])
        fig.colorbar(im, ax=axes[k], fraction=0.046, pad=0.04)

    for ax in axes[num_modes:]:
        ax.axis('off')

    plt.tight_layout()
    plt.show()

def square_spectrum_analytic(n_display):
    """
    Compute the n_display smallest exact eigenvalues of the Helmholtz equation
    on the unit square [0,1]x[0,1] with Dirichlet boundary conditions.

    Eigenvalues are  _{p,q} =  ²(p² + q²), with p,q >= 1.

    Parameters
    ----------
    n_display : int
        Number of smallest eigenvalues to return.

    Returns
    -------
    result : dict
        {
            "eigenvalues": ndarray of shape (n_display,),
            "indices": list of tuples (p, q) for each eigenvalue
        }
    """
    # Generate a pool of candidates (p, q up to some reasonable limit)
    limit = int(np.ceil(np.sqrt(n_display) * 3))  # overshoot to ensure enough modes
```

```
    candidates = []

    for p in range(1, limit + 1):
        for q in range(1, limit + 1):
            lam = np.pi**2 * (p**2 + q**2)
            candidates.append((lam, (p, q)))

    # Sort by eigenvalue
    candidates.sort(key=lambda x: x[0])

    # Take first n_display entries
    eigenvalues = np.array([c[0] for c in candidates[:n_display]])
    indices = [c[1] for c in candidates[:n_display]]

    return {
        "eigenvalues": eigenvalues,
        "indices": indices
    }
```

[2]:

```
result_exact = square_spectrum_analytic(12)

# print table
print("{:>5} {:>6} {:>16}".format("idx","(q,p)","lambda"))
for i, ((p,q), lam) in enumerate(zip(result_exact["indices"],␣
 ↪result_exact["eigenvalues"])):
    print(f"{i:5d} ({q},{p}) {lam:20.12f}")
```

```
  idx  (q,p)           lambda
    0 (1,1)      19.739208802179
    1 (2,1)      49.348022005447
    2 (1,2)      49.348022005447
    3 (2,2)      78.956835208715
    4 (3,1)      98.696044010894
    5 (1,3)      98.696044010894
    6 (3,2)     128.304857214162
    7 (2,3)     128.304857214162
    8 (4,1)     167.783274818519
    9 (1,4)     167.783274818519
   10 (3,3)     177.652879219608
   11 (4,2)     197.392088021787
```

[11]:

```
# Compute spectrum for unit square
result_square = square_spectrum_fd(N=101, M=12)

print("Eigenvalues:", result_square["eigenvalues"])

# Plot first 12 modes
```

```
plot_square_eigenfunctions(result_square, num_modes=12, indices =
    result_exact["indices"])
```

```
Eigenvalues: [ 19.73758537  49.33422412  49.33422412  78.93086287  98.62950062
  98.62950062 128.22613937 128.22613937 167.5747664  167.5747664
 177.52141588 197.17140515]
```

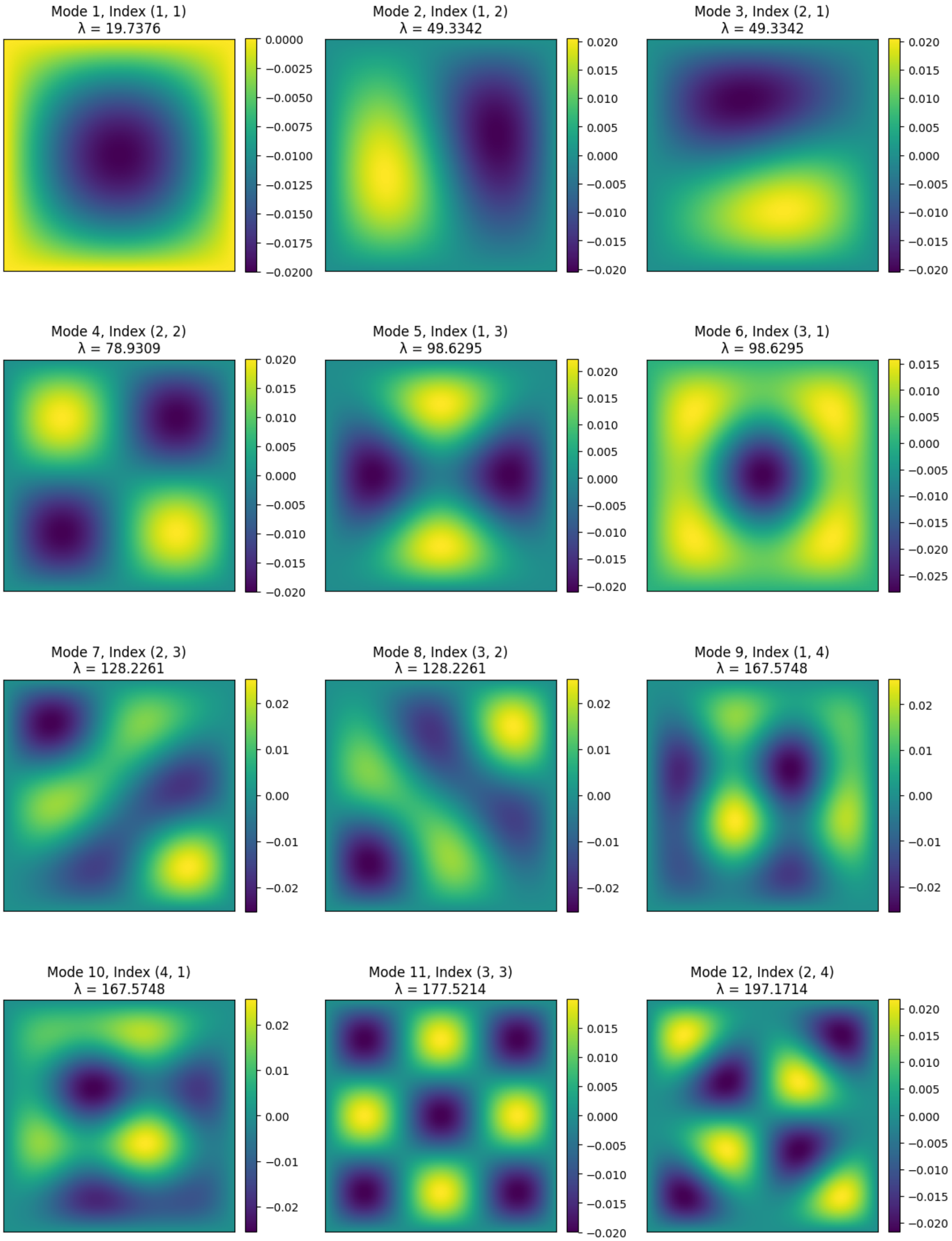

[4]:
```
# Number of first eigenvalues that we want to study
M_evals = 12

# Compute analytical spectrum of M_evals eigenvalues
sp_ana = square_spectrum_analytic(M_evals)
sp_ana_vals = sp_ana["eigenvalues"]

# Compute relative difference of eigenvalues as the grid is refined
delta_spectrum = []
for N_grid in range(10,150):
    sp_num_vals = square_spectrum_fd(N = N_grid, M = M_evals)["eigenvalues"]
    delta_spectrum.append((N_grid, (sp_num_vals - sp_ana_vals)/sp_ana_vals))
```

[5]:
```
# Transform results for easier plotting
res_transf = np.array([[(N_grid, v) for v in vals] for N_grid, vals in␣
 ↪delta_spectrum])
res_transf = np.swapaxes(res_transf,0,1)
```

[6]:
```
fig, ax = plt.subplots(figsize = (10, 7))

# Separately plotting for each eigenvalue
for i, r in enumerate(res_transf):
    idx = sp_ana["indices"][i]
    ax.plot(r[:,0],-r[:,1], "o-", label = f"(q,p)=({idx[0]},{idx[1]})", alpha=0.
 ↪7)

# adding rough dependence
x = np.linspace(10, 200, 100)
ax.plot(x, x**-2, lw = 2, label = r"$N_{grid}^{-2}$")

ax.set_xlabel(r"$N_{grid}$")
ax.set_ylabel(r"$1 - \lambda_{i, num}/\lambda_{i,ana}$")

ax.set_xscale("log")
ax.set_yscale("log")

ax.legend(loc='center left', bbox_to_anchor=(1, 0.5))
plt.show()
```

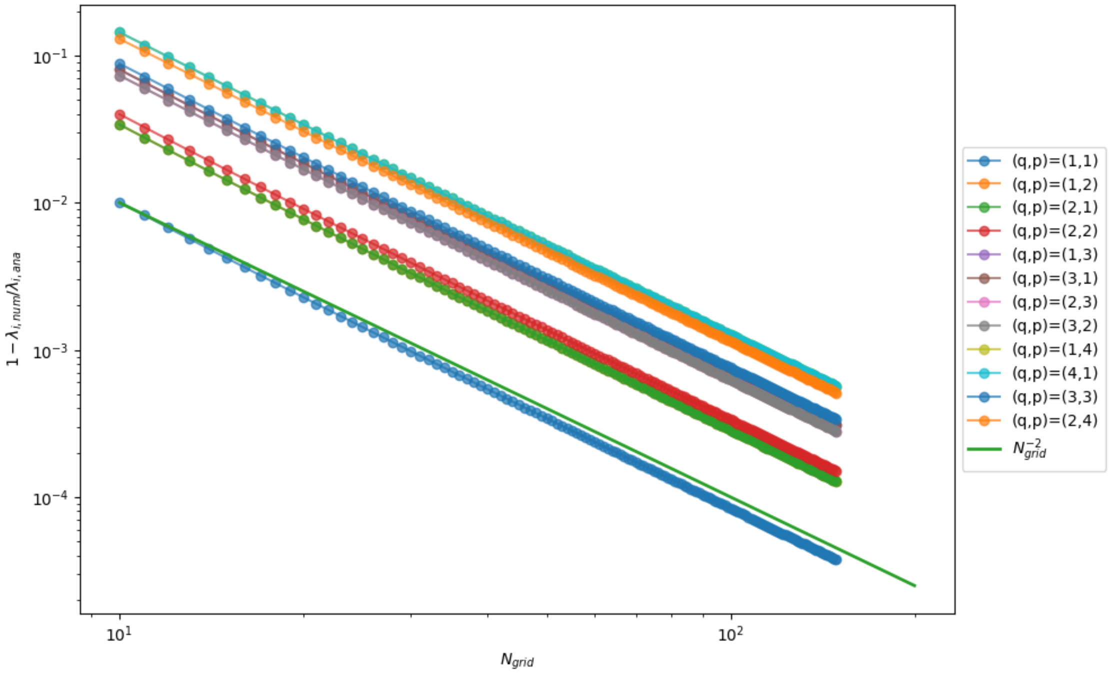

[7]:
```
fig, ax = plt.subplots(figsize = (10, 7))



# Plotting relative difference of near degenerate pairs
idx0 = (None, None)
for i, r in enumerate(res_transf):
    idx = sp_ana["indices"][i]

    if (idx[0] == idx0[1]) and (idx[1] == idx0[0]):
        ax.plot(r[:,0], np.abs(rold - r[:,1]), "o-", label = f"(n,m) =␣
 ↪({idx[0]},{idx[1]})", alpha=0.7)
    else:
        rold = r[:,1]

    idx0 = idx[:]


ax.set_xlabel(r"$N_{grid}$")
ax.set_ylabel(r"$|(\lambda_{\mathrm{first in pair, num}} -␣
 ↪\lambda_\mathrm{second in pair, num})|/\lambda_{i,ana}$")

ax.set_title("Relative difference of near degenerate pairs")

ax.set_xscale("log")
```

```python
ax.set_yscale("log")

ax.legend(loc='center left', bbox_to_anchor=(1, 0.5))
plt.show()
```

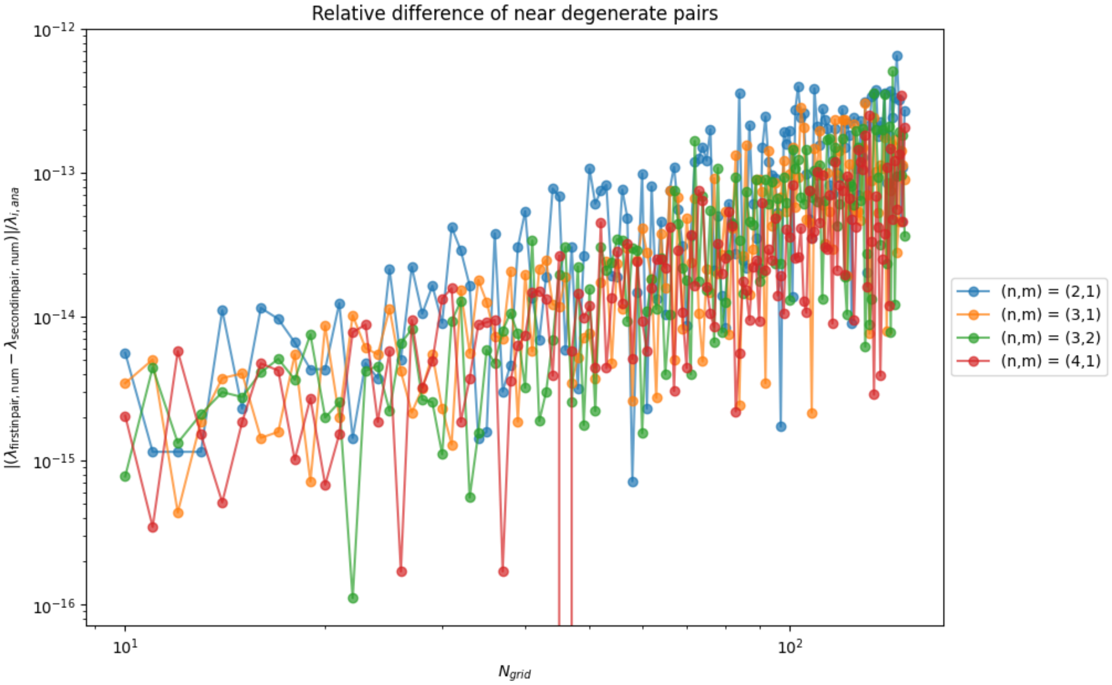

Conclusion:

We can see that we have numerically exact degeneracy with multiplicity of 2 for all $q \neq p$. The discrepancy of eigenvalues from exact ones goes as $(O(h^{-2}))$ and clearly dominated by Laplacian operator discretization error.
