# Supplementary figures and images for: Photonic Eigenmodes of 2D Cylindrical Cholesteric Liquid Crystal Resonators
Source: ACS Photonics. 2025 Sep 26;12(10):5572–85. doi: 10.1021/acsphotonics.5c01294 (PMC12532369; doi:10.1021/acsphotonics.5c01294)

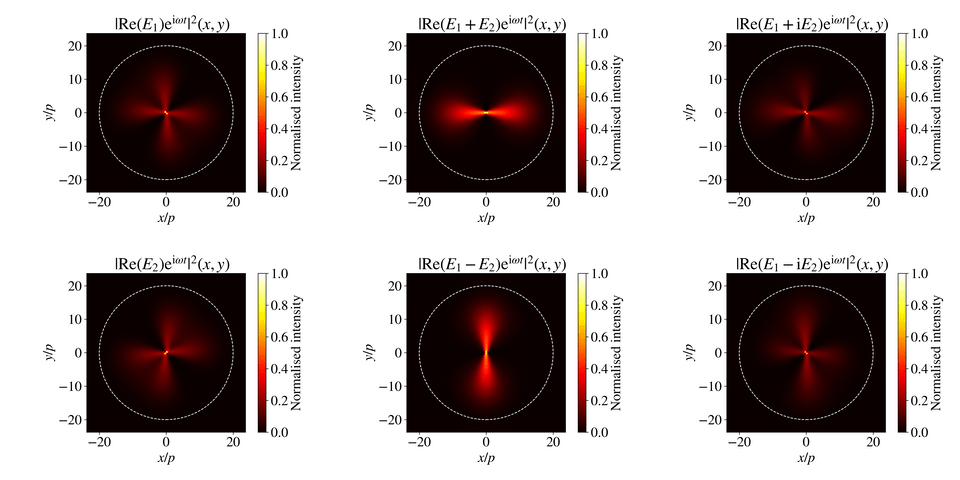

Supplement: Supplementary file 4 [file ph5c01294_si_004.gif]

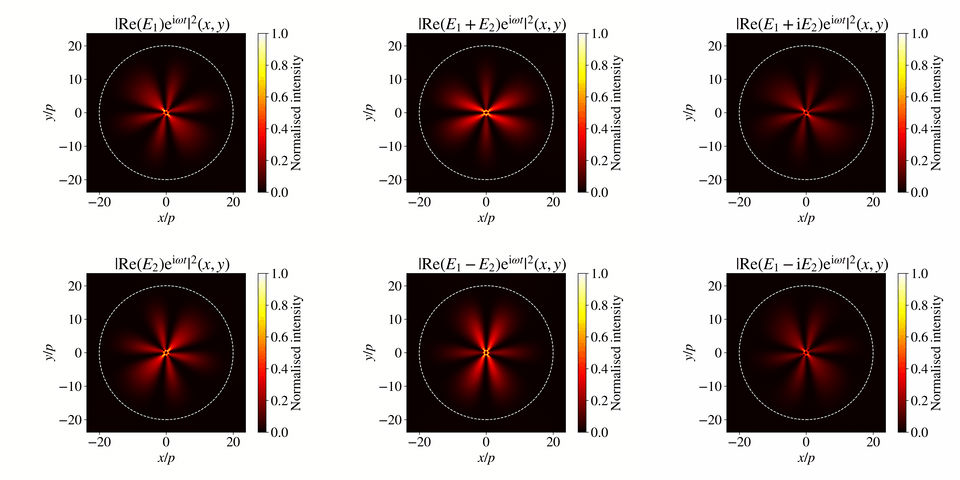

Supplement: Supplementary file 5 [file ph5c01294_si_005.gif]
